# Calcium inhibits penetration of Alzheimer's Aβ_1_–_42_ monomers into the membrane

**DOI:** 10.1002/prot.26403

**Published:** 2022-08-10

**Authors:** Subramanian Boopathi, Ramón Garduño‐Juárez

**Affiliations:** ^1^ Instituto de Ciencias Físicas Universidad Nacional Autónoma de México Cuernavaca Mexico

**Keywords:** Alzheimer disease, Aβ_1‐42_, Aβ_1–42_‐Cu^2+^, calcium ions, DMPC bilayer, microsecond MD

## Abstract

Calcium ion regulation plays a crucial role in maintaining neuronal functions such as neurotransmitter release and synaptic plasticity. Copper (Cu^2+^) coordination to amyloid‐β (Aβ) has accelerated Aβ_1–42_ aggregation that can trigger calcium dysregulation by enhancing the influx of calcium ions by extensive perturbing integrity of the membranes. Aβ_1–42_ aggregation, calcium dysregulation, and membrane damage are Alzheimer disease (AD) implications. To gain a detail of calcium ions' role in the full‐length Aβ_1–42_ and Aβ_1‐42_‐Cu^2+^ monomers contact, the cellular membrane before their aggregation to elucidate the neurotoxicity mechanism, we carried out 2.5 μs extensive molecular dynamics simulation (MD) to rigorous explorations of the intriguing feature of the Aβ_1–42_ and Aβ_1–42_‐Cu^2+^ interaction with the dimyristoylphosphatidylcholine (DMPC) bilayer in the presence of calcium ions. The outcome of the results compared to the same simulations without calcium ions. We surprisingly noted robust binding energies between the Aβ_1–42_ and membrane observed in simulations containing without calcium ions and is two and a half fold lesser in the simulation with calcium ions. Therefore, in the case of the absence of calcium ions, N‐terminal residues of Aβ_1–42_ deeply penetrate from the surface to the center of the bilayer; in contrast to calcium ions presence, the N‐ and C‐terminal residues are involved only in surface contacts through binding phosphate moieties. On the other hand, Aβ_1–42_‐Cu^2+^ actively participated in surface bilayer contacts in the absence of calcium ions. These contacts are prevented by forming a calcium bridge between Aβ_1–42_‐Cu^2+^ and the DMPC bilayer in the case of calcium ions presence. In a nutshell, Calcium ions do not allow Aβ_1–42_ penetration into the membranes nor contact of Aβ_1–42_‐Cu^2+^ with the membranes. These pieces of information imply that the calcium ions mediate the membrane perturbation via the monomer interactions but do not damage the membrane; they agree with the western blot experimental results of a higher concentration of calcium ions inhibit the membrane pore formation by Aβ peptides.

**Abbreviations**
AD: Alzheimer disease
Aβ: β‐amyloid peptide
DMPC: dimyristoylphosphatidylcholine
MD: molecular dynamics
Cu^2+^
: Copper
Ca^2+^
: Calcium

## 1 INTRODUCTION

Over 50 million people succumbed to Alzheimer disease (AD) worldwide is expected to double every 20 years, reaching 75 million in 2030 and 131.5 million in 2050 unless scientists predict effective therapeutic strategies.^1^ AD is the most prevalent cause of dementia, accounting for up to 80% of all dementia diagnoses and is characterized by the formation of amyloid β (Aβ) plaques constituted by the aggregation of Aβ peptides, which are produced from the transmembrane amyloid precursor protein (APP) after being cleaved by β‐ and γ‐secretases.^2^ In the case of the healthy brain, Aβ peptides are in monomeric forms soluble in nature. In contrast, in the case AD affected brain, the peptides aggregate into soluble oligomers and then insoluble fibrils^3^ resulting in the formation of plaques.^4^ Aβ_1–42_ exerts more hydrophobicity in comparison to Aβ_1–40_, driving aggregation‐prone structure; thus, Aβ_1–42_ is the abundant species in senile plaques.^5^ The soluble oligomers contribute to several events in AD pathogenesis, including membrane permeability, mitochondrial damage, oxidation stress, and calcium dysregulation.^6^, ^7^

Using in silico, in vitro, and in vivo experiments, we found^7^ that Aβ_1–42_ peptides triggered neurotoxicity, synaptic toxicity, calcium dyshomeostasis, and memory impairment in AD mice brains. Although a large number of experiments^8^ have addressed the interaction of oligomeric and fibrils of Aβ peptides with model membranes and some of the theoretical investigations^9^, ^10^, ^11^, ^12^, ^13^, ^14^ have studied the monomers, oligomers, and fibril with close contact with the membranes; so far, the underlying mechanism has not been fully elucidated. Still, the interaction of peptides with the membranes is an essential to unveiling neurotoxicity. The interaction of the Aβ peptide with the membrane bilayer depends upon the peptide concentration. For instance, the monomeric peptides have been found binding to the membrane at lower concentration (≤150 nM),^13^ while the higher peptide concentration induced oligomeric peptide interaction with the membrane.^12^ In particular, small oligomer rather than larger aggregates exerts elevated binding affinity with the membrane.^15^

Elevated concentrations of copper ions (400 μM) have been observed in the postmortem AD brain and associated with several neurodegenerative disordered.^16^ Experiments^17^, ^18^, ^19^ have given rise to the hypothesis that Cu^2+^ ions bound to Aβ_1–42_ peptides are involved in three main intertwined pathological events to neuronal cell death; (a) overproducing reactive oxygen species (ROS) contributing to oxidation stress, (b) inducing Aβ_1–42_ peptides aggregation mediates neuronal damage by forming membrane perforation, and (c) an enhancement in intracellular calcium levels. The toxicity induced by Aβ_1–42_‐Cu^2+^ peptides aggregations is well correlated with peptide–membrane interactions.^20^

To characterize the effect of copper ions in the Aβ aggregation process, in our two previous papers^21^, ^22^ we elucidated that Cu^2+^ binding promotes a higher solvation free energy (more hydrophobic) in Aβ_1–42_ peptides. The greater water‐mediated attraction propensity dictates the fastest self‐assembly of Aβ_1–42_‐Cu^2+^ compared to Aβ_1–42_. However, previous simulations of Aβ_1–42_ and Aβ_1–42_‐Cu^2+^ monomers were modeled aqueous phase; their behavior in the cellular environment is still lacking, which is essential to revealing the toxicity mechanism that has been shown to Aβ oligomers' direct contact with the neuronal membranes.

On the other hand, in 1989,“calcium hypothesis of brain aging proposed^23^ that Ca^2+^ ions are indispensable element for brain function contributing to neurotransmission release, synaptic plasticity, and gene expression. The uncontrollable in and out Ca^2+^ transport of cellular membranes occurred in the AD brain induced by Aβ aggregation in hippocampal neurons. This effect mediated cognitive dysfunctions by generating neuroinflammation, synaptic failure, neurotoxicity, and synaptic plasticity.^24^ Thus, the relationship between Aβ and Ca^2+^ ions reinforce the cognitive deficits in AD patients.

Mounting evidence^19^, ^25^, ^26^, ^27^, ^28^, ^29^ envisaged the membrane attracted Aβ peptides through electrostatic interaction. Subsequently, the peptides penetrating the membrane were driven by hydrophobic interaction of the peptides' central hydrophobic and C‐terminal residues with the membranes. Many studies^19^, ^30^, ^31^, ^32^ have been intensively investigated the consequence of free and copper‐bound Aβ peptide's interaction with the membrane. Notably, experimental^33^, ^34^, ^35^ and computational^36^, ^37^, ^38^, ^39^ evidence accounted that calcium ions strongly interact with zwitterionic phosphatidylcholine lipid bilayers. One possible mechanism expects that calcium ions influence these peptides' binding to the zwitterionic lipid bilayer closely related to neuronal toxicity. Exploring the interaction of free and copper‐bound full‐length Aβ_1–42_ monomeric peptides with the membrane in the presence of Ca^2+^ concentration is an initial step to identifying the aggregation and cytotoxicity that is still elusive. However, capturing these transition interactions at the atomistic level is challenging with the experimental method. Thus, to best of our knowledge, we first address this problem in the present work by employing rigorous explicit water microsecond molecular dynamics (MD) simulations.

MD simulations of Aβ_1–42_ and Aβ_1–42_‐Cu^2+^ monomers interacting with zwitterionic DMPC bilayer in the presence and absence^40^ of calcium ions concentration. In the case of the absence of calcium ions, N‐terminal residues of Aβ_1–42_ deeply penetrate from the surface to the center of the bilayer, and C‐terminal of residues of Aβ_1–42_‐Cu^2+^ can participate in surface penetration by binding phosphate moieties.^40^ In the case of the presence of calcium ions, our present simulation results revealed that N‐terminal and C‐terminal residues of Aβ_1–42_ were involved in surface contacts by binding phosphate moieties; these contacts disappeared in the case of Aβ_1–42_‐Cu^2+^. These observations imply that Ca^2+^ ions play a significant role in preventing the Aβ_1–42_ peptide penetration into the membrane and inhibiting contact of Aβ_1–42_‐Cu^2+^ with the membrane.

## 2 COMPUTATIONAL METHOD

### 2.1 Simulation set up

**FIGURE 1:**
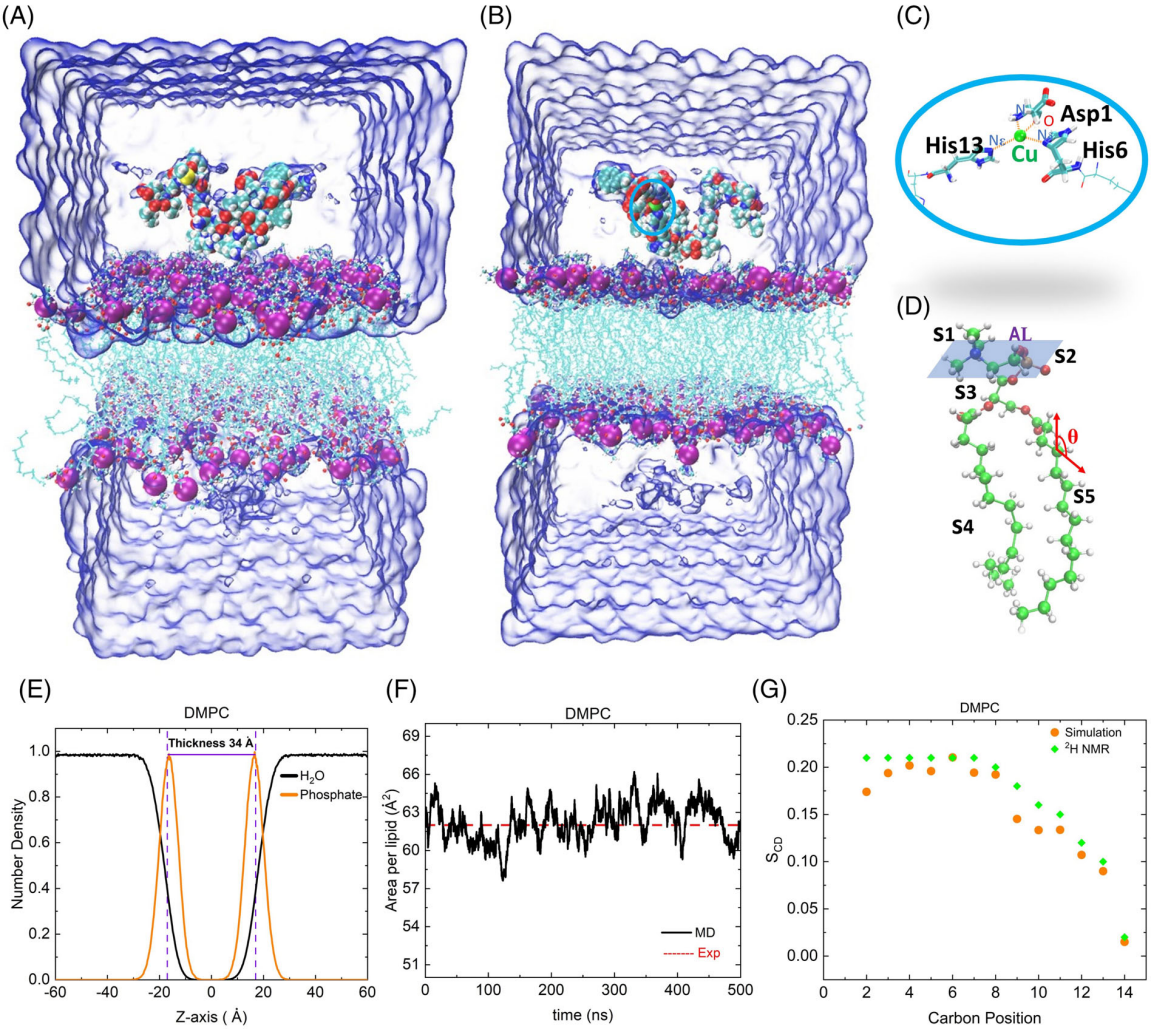
(A) Initial geometry for simulation containing DMPC bilayer, full‐length of Aβ_1–42_ (van der Waals representations), and water (blue) and CaCl_2_ ions (hidden for clarity purpose) and (B) Cu^2+^ bound full‐length Aβ_1–42_ peptide (van der Waal representation), C, N, O, S, H, and Cu atoms shown in cyan, blue, red, yellow, white and green, respectively; phosphorous atoms shown in purple. (C) Metal coordination residues highlighted, Cu^2+^ (green ball) coordinates to N and O of Asp1, Nδ of His6, and Nε of His13. (D) The bilayer constituted by choline (S1), phosphate (S2), glycerol (S3), and fatty acids tails (S4 and S5); area per lipid shown in a blue plane and the angle along one of the lipid acyl chains represented by red. (E) Normalized number density profile for the bilayer and the water, and (F) area per lipid plotted against time and red dotted line indicated experimental^41^, ^42^ observed value. (G) Simulation NMR order parameters of the acyl chains of bilayer and comparison to experimental data^43^

We have performed extensive MD simulations to characterize the Aβ_1–42_ and Aβ_1–42_‐Cu^2+^ peptide dynamics on DMPC lipid membranes coincubated with Ca^2+^ ions (Figure 1A,B); simulation details are tabulated in Table 1. Man et al.^44^ have demonstrated that the five force fields, AMBER99SB‐ILDN, AMBER14SB, CHARMM22*, CHARMM36, and CHARMM36m, are the best candidates among 17 atomic molecular‐mechanics force fields for Aβ peptide studies. Subsequently, Krupa et al.^45^ reported results from CHARMM36m and AMBERFF14SB MD simulations on Aβ peptide, which corroborated with experimental data. When the peptides and membranes were in the simulation, AMBERFF14SB and LIPID14 rendered optimal accuracy for the lipids‐peptides systems because the primary force field gives a similar result to the latter one.^46^ Thus, in the present simulation, we applied AMBERFF14SB for the peptide, calcium chloride, and TIP3P water model and LIPID14 for the DMPC lipid membranes in the present simulation report.

**TABLE 1:** Summary of molecular dynamics simulation details

Simulation	Composition	Replicas	Time (ns)	Box dimension (Å)	Number of water
Aβ_42_/DMPC	Aβ_42_ + 154 DMPC + 80 Ca^2+^ + 157 Cl^−^	5	500	74.88 × 74.37 × 124.39	13 332
Aβ_42_‐Cu^2+^/DMPC	Aβ_42_‐Cu^2+^ + 154 DMPC + 80 Ca^2+^ + 159 Cl^−^	5	500	74.88 × 74.37 × 124.39	13 330

Abbreviation: DMPC, dimyristoylphosphatidylcholine.

### 2.2 Molecular dynamics simulation protocol

The bonded model of the full‐length of Aβ_1–42_‐Cu^2+^ was taken from our previous MD simulation,^47^ where Cu^2+^ is coordinated to nitrogen and oxygen atoms of Asp1, N_δ_ of His6, and N_ε_ of His13 (Figure 1C). Huy et al.^47^ have reported force‐field parameters between Cu^2+^ and the coordination atoms used in the present work (see supporting information S1). The Aβ_1–42_ peptide contains three positively charged residues (Arg5, Lys16, and Lys28), six negatively charged residues (Asp1, Glu3, Asp7, Glu11, Glu22, and Asp23), and net charged is −3. The Aβ_1–42_‐Cu^2+^ is −1 because of N‐terminal deprotonation. The Aβ_1–42_ and Aβ_1–42_‐Cu^2+^ peptides were separately immersed into a simulation box of dimension 74.88 Å × 74.37 Å × 124.39 Å containing 13 330 water molecules, 200 mM concentration of Ca^2+^ ions, and 144 lipid bilayers (Figure 1D) in which each bilayer leaflet has 77 DMPC lipids arranged in square shape. Notably, the extracellular Ca^2+^ concentrations in the brain are 1–2 mM, which accounts for the presence of four calcium ion in our present simulation box size 679 024 Å^3^. Therefore, we performed molecular dynamics simulations by adding a much higher calcium concentration in the simulation box. The center of mass of phosphorous (P) atoms in each leaflet is ±Zp = ~17 Å from midplane Z = 0, and the thickness of the bilayer was ~34 Å (*D* = 2Zp) (Figure 1E). Several chloride ions were added to neutralize the system, and the net charge of the simulation system was zero. Four significant points were motivated to select the DMPC bilayer: (a) this lipid is abundant in the neuronal cell membrane^48^; (b) it is a shorter chain length than the average human lipid membrane length, and structural and physicochemical properties are well documented in the literature^49^, ^50^; (c) experiments^49^ demonstrated that the behavior of lipid order of DMPC as a function of temperature was closer to human body physiological conditions of 37°C and 310 K; and (d) NMR spectroscopy data^51^ robustly suggested that this bilayer is a potential candidate for exploring the interaction mechanism between amyloid peptide and the membrane. In addition, Lockhart and Klimov^29^, ^52^ have demonstrated that experimental observation of binding of Aβ with the DMPC bilayer for SDS micelles^53^, ^54^ was direct compared to simulation results.

Five hundred steps of steepest descent minimization, followed by 500 steps of conjugate gradient method, were applied to energy minimize each system with the peptide constrained by applying a force of 100 kcal/mol‐Å^2^. The systems were further minimized using the same procedure without restraints. Multiple microsecond‐length production runs using an isobaric‐isothermal (NPT) statistical ensemble at the constant temperature (300 K) and pressure (1 atm). The Langevin thermostat^55^ was applied to control the temperature with a collision frequency of 2 ps^−1^, the Berendsen barostat to maintain the pressure with a relaxation time of two picoseconds, and the SHAKE algorithm^56^ to constrain bonds involving hydrogen atoms. The Particle Mesh Ewald algorithm^57^ was employed to compute the long‐range electrostatic and van der Waals interaction. We calculated nonbonded interactions with a fixed cut‐off of 10 Å; the simulation time‐step was 2 fs. We collected five trajectories of each peptide after starting a simulation of each system at different initial velocities, and the corresponding analysis was carried out based on five 500 ns‐trajectories. A total of 2.5 μs (5‐trajectories × 500 ns) simulation of Aβ_1–42_/DMPC and Aβ_1–42_–Cu^2+^/DMPC complexes were sufficient (see Supporting Information S1) to unveil the interaction mechanism between the peptides and DMPC bilayers in the presence of a high concentration of Ca^2+^ ions in terms of structural and thermodynamics description.

### 2.3 Structural details

A DMPC lipid molecule was divided into choline (S1), phosphate (S2), glycerol (S3), and two fatty acid tail (S4 and S5) regions, as shown in Figure 1D. To understand the Aβ_1–42_ peptide interaction mechanism with the membrane, the peptide was divided into four regions: hydrophilic N‐terminal (NT R1, residues 1–16), central hydrophobic region (CHR R2, residues 17–21), loop region (LR R3, residues 22–28), and hydrophobic C‐terminal (CT R4, residues 29–42), the peptide sequence is indicated by one letter amino acid code as follows:

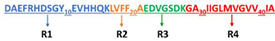

where blue, orange, green, and red‐colored letters represented R1, R2, R3, and R4 segments, respectively.

### 2.4 Analysis

Production trajectories were analyzed using the cpptraj program of AMBER16 packages,^58^ which was used to obtain root‐mean‐square deviation (RMSD), secondary structure, solvent accessible surface area, hydrogen bonds pattern, and density of mass analyses. We wrote Perl scripts to analyze the contact map and free‐energy calculation (see Supporting Information S1).

#### 2.4.1 Root mean square deviation

The RMSD^59^ of the Cα atoms of Aβ_1‐42_/DMPC and Aβ_1–42_‐Cu^2+^/DMPC was calculated for five 500 ns replicas.

#### 2.4.2 Secondary structure

Defined secondary structure of protein (DSSP) method^60^ program in AmberTools 16 was utilized to calculate the peptides' secondary structure along with the time of the MD simulations. The averages of secondary structural contents are denoted by <…> and beta sheets (sum of parallel and antiparallel), helix (sum of α, 3_10_ and ᴫ), turn and random coil (unstructured conformer) represented as <BS>,<H>,<T>,and<RC>, respectively.

#### 2.4.3 Contact map

We mapped the contact between two residues when the distance between the center of masses of any two residues is below 6.5 Å. In the case of membrane and peptide interactions, contact was considered between these entities when the distance between the center of masses of amino acids and one of the lipid groups (S1–S4) was ≤6.5 Å.

#### 2.4.4 Solvent accessible surface area

The LCPO method^61^ in the cpptraj program was used to determine the solvent‐accessible surface area (SASA) per residue, where the calculation is based on the spherical surface around each residue atom with a distance of 1.4 Å away from the atoms of the van der Waals surface.

#### 2.4.5 Hydrogen bonds

Hydrogen bonds were considered when X—Y distance in X—H…Y is small than 3.5 Å and X—H…Y angle is larger than 135^°^.

#### 2.4.6 Salt bridges

Salt bridges were calculated between positively charged amino acids (Arg5, Lys16, and Lys28) and negatively charged residues (Asp1, Glu3, Asp7, Glu11, and Asp23). Ensuing Equation (1) measures the salt bridges.

$$\mathrm{SB}=\sum_{i,j}S_{i,j}$$

$$S_{i,j}=1\;\text{if}\;r_{i,j}\leq 0$$

$$S_{i,j}=0\;\text{if}\;r_{i,j}>0$$

(1) $$r_{i,j}=\left|r_{i}-r_{j}\right|-d_{0}$$

where *i* and *j* are running over different sets of atoms pairs, each pair contains a different portion of the system, we inspected the intramolecular salt bridge between charged amino acids by selecting two‐atom sets, one atom from the positively charged group of N_ε_(Lys) and N_η_(Arg), and another atom from the negative group of C_γ_(Asp) and C_δ_(Glu). *d*_0_ is the distance between atoms *i* and *j*. The value of *d*_0_ was 4.5 Å.

#### 2.4.7 Area per lipid and NMR order parameter

The area per lipid (A_L_) or in‐plane area occupied by a given lipid (see blue color in Figure 1D) was measured using the following equation:

(2) $$A_{L}=\frac{L_{X}L_{Y}}{n}$$

where *L*_*x*_ and *L*_*y*_ are the lateral dimensions of the simulation box along the x and y axes, respectively; and $n=\frac{N_{L}}{2}$ is the number of lipids per leaflet.

Deuterium NMR order parameter (S_CD_) describes the lipid arrangement within the membrane. One C—H bond vector is shown in the lipid tail. we measure the orientation of vector (red in Figure 1D) with respect to z‐axis (bilayer normal) for determine S_CD_ by using the following equation .

(3) $$S_{\mathrm{CD}}=\frac{1}{2}\left\langle 3{\mathrm{COS}}^{2}\theta -1\right\rangle$$

where *θ* is angle between the C—H bond vector and the bilayer normal; the angular brackets represent the ensemble average.

#### 2.4.8 Density of mass analysis

The density of mass for peptides, membranes, and ions was calculated using the density tool in the cpptraj program. Lipid bilayer thickness is determined by measuring the distance between the density of the phosphorous atoms in the upper and lower leaflets.

#### 2.4.9 Binding free energy

The molecular mechanics‐generalized Boltzmann surface area (MM‐GBSA)^62^ python script along with AMBER16 was used to compute the binding free energy between the peptide and membrane. The snapshots were collected at 100 ps intervals over the 500 ns MD trajectories, and the MM‐GBSA calculations were carried out using the following equation:

$$∆G_{\text{bind}}=∆E_{\mathrm{MM}}+∆G_{\text{solv}}-T∆S$$

$$∆E_{\mathrm{MM}}=∆E_{\mathrm{int}}+∆E_{\text{elec}}+∆E_{\mathrm{vdw}}$$

(4) $$∆G_{\text{solv}}=∆G_{\mathrm{GB}}+∆G_{\mathrm{SA}}$$

Total binding free energy (Δ*G*_bind_) is the sum of the gas‐phase interaction between peptide and the membranes (Δ*G*_MM_), the solvation energy associated with the transition from the gas‐phase to the solvated state (Δ*G*_solv_), and the changes in conformational entropy associated with membrane binding (−TΔS). The internal, electrostatic, and van der Waals interaction energies are denoted as Δ*E*_int_, Δ*E*_elec_, and Δ*E*_vdw_, respectively. The polar contribution to the solvation free energy is determined using the Poisson–Boltzmann implicit solvent model (Δ*G*_PB_), and nonpolar contribution is measured based on the solvent‐accessible surface area (Δ*G*_SA_). MM‐GBSA differs from the molecular mechanics‐ poison Boltzmann surface Area (MM‐PBSA) in the use polar solvation free energy term Δ*G*_GB_ instead of ΔG_PB_. The Wang et al.'s review^63^ addressed three significant points: (a) MM‐PBSA can render lower accuracy of prediction of bonding radii for more extended simulation (>1 ns), (b) MM‐GBSA and QM‐MM/GBSA provided better binding affinity compared to MM‐PBSA for using multiple independent trajectories and long simulation method, and (c) MM‐GBSA estimated binding affinity in best agreement with the experimental binding results; therefore, we computed binding free energy for the peptide‐membrane complex by using MM‐GBSA method (Equation 2).

#### 2.4.10 Free energy landscape

Free energy surface (FES)^64^ of the systems using two reaction coordinates, V = (Rg, RMSD), was computed with Equation (5).

(5) $$G\left(V\right)=-K_{B}T\left[\ln P\left(V\right)-\ln P_{\max}\right]$$

where *P*(*V*) is the probability distribution obtained from the MD simulation results and *P*_max_ is the maximum of the distribution.

## 3 RESULTS

### 3.1 DMPC bilayer is in liquid‐ordered phase

Before discuss the Aβ‐membrane interaction, we determined the DMPC bilayer's characteristics that behave as a liquid order phase. We performed 500 ns MD simulation of the DMPC bilayer in the aqueous phase. The mass density profile of lipid bilayer and water along the membrane *z*‐axis is depicted in Figure 1E. The headgroup–headgroup distance of phosphocholine indicates a bilayer thickness of ~34 Å. Subsequently, we calculated the area per lipid and determined a value of 62.18 ± 1.45 ${\overset{{}^{\circ}}{A}}^{2}$ (Figure 1F) is in good agreement with the experimental^41^, ^42^ values ranging from 58.9 to 65.2 $\;{\overset{{}^{\circ}}{A}}^{2}$.

In addition, we monitor acyl chain arrangement within the membrane by measuring the order parameter S_CH_ of the C—H bonds of all the lipid tails (Figure 1G). These order parameter values are close to the deuterium NMR experimental data.^43^ Notably, these lipid properties values confirmed that the DMPC bilayer is the liquid‐ordered phase, which agrees with previous experimental observations.^46^, ^65^

**FIGURE 2:**
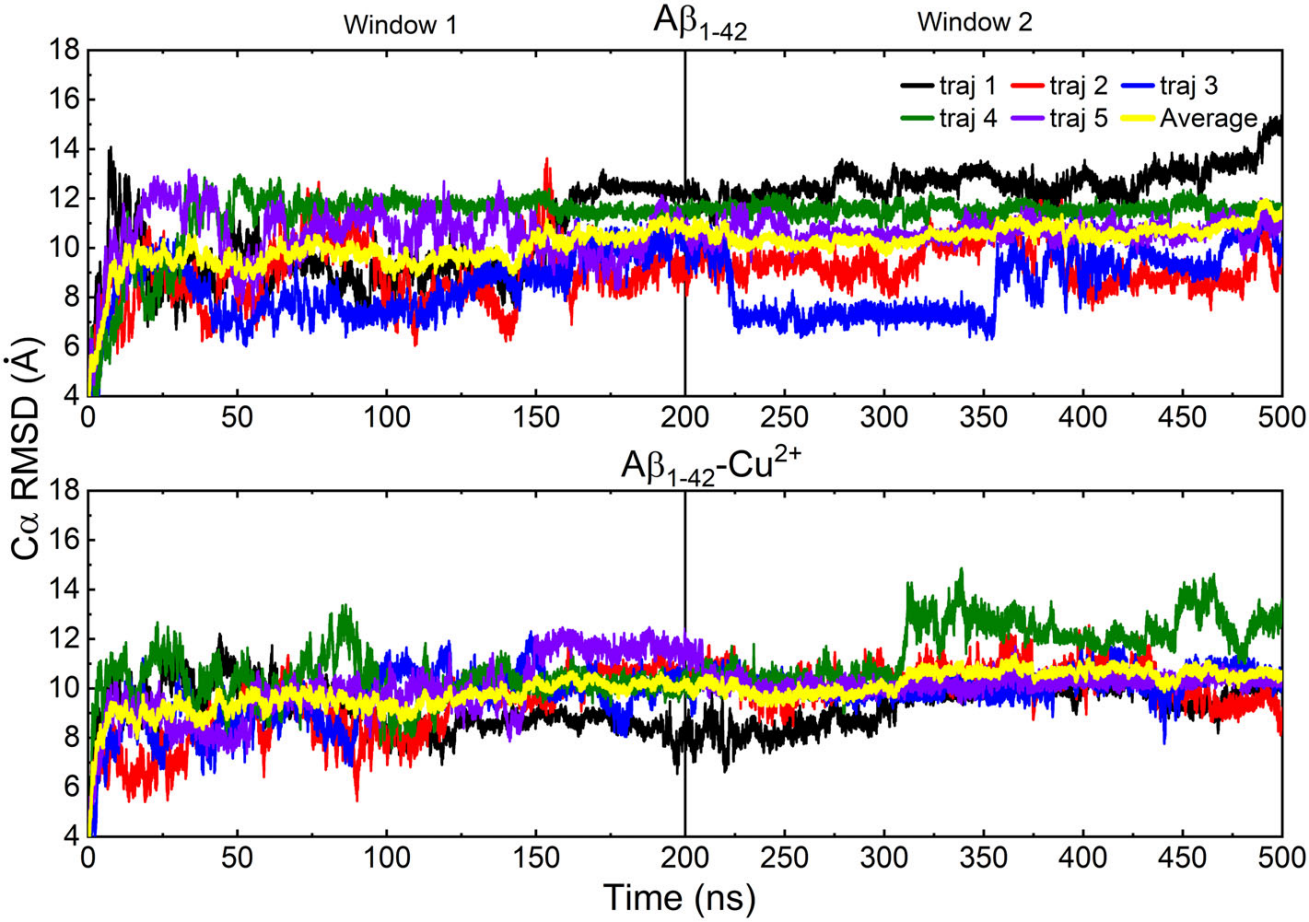
The Cα RMSD plotted against time for each trajectory of the Aβ_1–42_ and Aβ_1–42_‐Cu^2+^ over two‐time windows [200, 500 ns]

The averaged time‐dependent of the Cα rmsd, the radius of gyration (Rg), and total solvent‐accessible surface area (SASA) indicate that all the simulation systems became stable after 200 ns (Figures 2 and S1). Since the peptide‐membrane complex fluctuated around the equilibrium value after the *τ*_eq_ ≈ 200 ns, data analysis was carried out in the 200–500 ns range.

### 3.2 Cu^2+^ binding promotes higher β‐sheet contents in Aβ_1–42_ monomer

**FIGURE 3:**
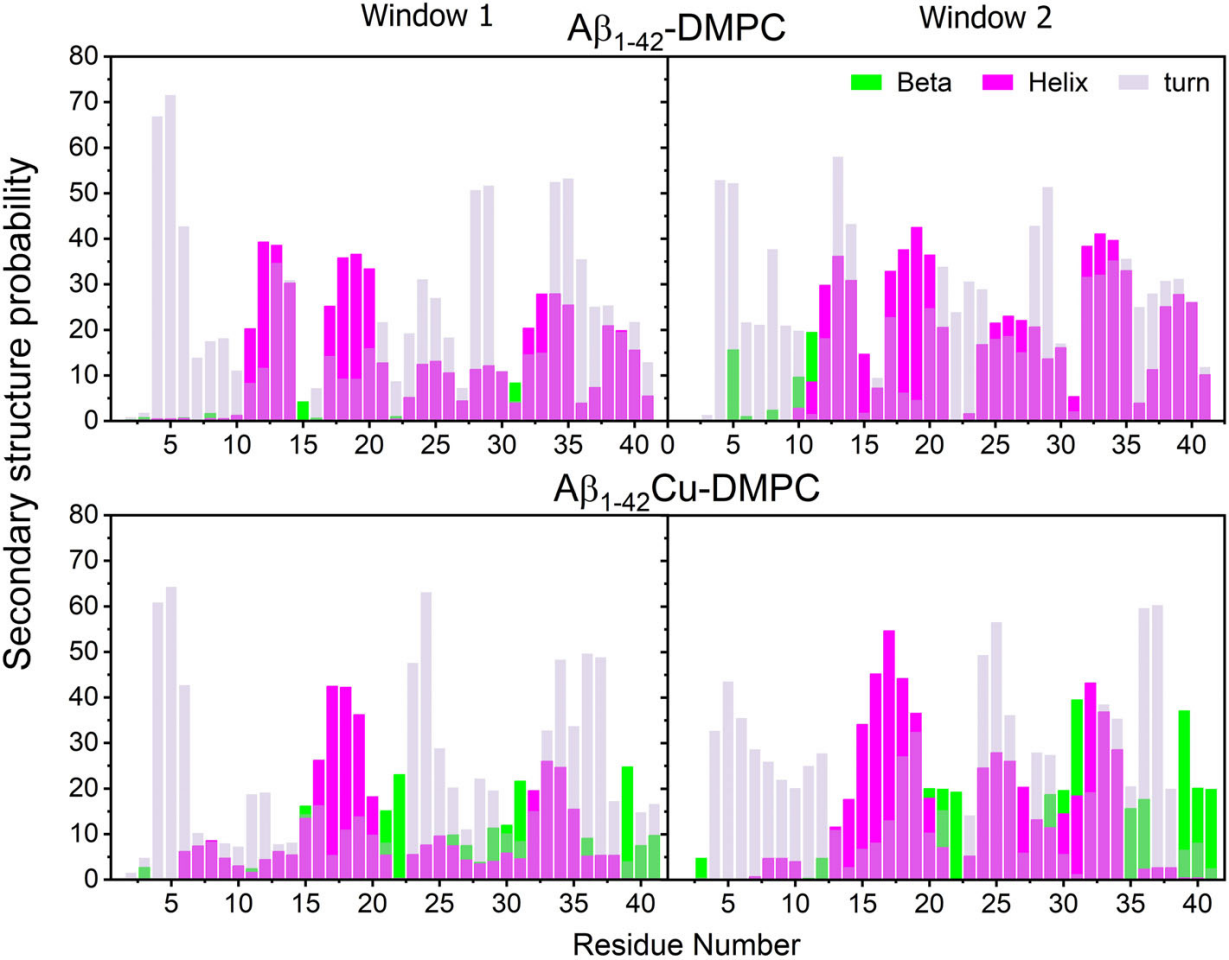
Residue‐wise population distribution (%) of the secondary structure of Aβ_1–42_ and Aβ_1–42_‐Cu^2+^ in the presence of dimyristoylphosphatidylcholine (DMPC) and Ca^2+^ ions. Analysis was carried out for an average of all trajectories over two‐time windows [200, 500 ns].

To evaluate the structural propensity of Aβ_1–42_ and Aβ_1–42_‐Cu^2+^ in close contact with the DMPC bilayers in the presence of Ca^2+^ ions (Table 2), we divided the peptide into four regions, R1 spans residues Asp1‐Lys16, R2 spans residues Leu17‐Ala21, R3 spans residues Glu22‐Lys28 and R4 spans residues Gly29‐Ala42. Average secondary structure contents presented in Table 2 shows beta‐sheet <BS>, helix <H>, turn <T>, and random coil <RC> of each region and entire peptide for Aβ_1–42_ and Aβ_1–42_‐Cu^2+^ complexes. Aβ_1–42_‐Cu^2+^ possessed higher <BS> (7.02% ± 2.50%) and lower <H > (13.39% ± 2.06%) compared to Aβ_1–42_ < BS> (1.71% ± 0.84%) and <H> (16.57% ± 0.87%). Importantly, in comparison to Aβ_1–42_, R4 has provided significant contribution in Aβ_1–42_‐Cu^2+^ secondary structures propensity by increasing <BS> 12.73% and decreasing <H> 9.22%; and in addition, Cu^2+^ ion decreasing 6.18% of <H> in R2 of Aβ_1–42_. Our findings imply that Cu^2+^ binding causes two significant changes in Aβ_1–42_/DMPC complex (Figure 3), (a) <BS> formation in R2 and R4 containing residues and (b) disappearing the <H> contents in residues Met35‐Ile41 and converting them into <BS>; therefore, Cu^2+^ binding mediate to enhancing <BS> contents in entire Aβ_1–42_/DMPC complex by reducing <H> contents.

**TABLE 2:** Average secondary structure content (%) ± standard error

System	Structure	R1	R2	R3	R4	Peptide
Aβ_42_/DMPC	<BS>	3.13 ± 1.91	1.76 ± 1.75	0.73 ± 0.68	0.74 ± 0.41	1.71 ± 0.84
	<H>	8.13 ± 2.40	33.94 ± 16.81	15.07 ± 10.63	20.77 ± 2.35	16.57 ± 0.87
	<T>	22.40 ± 3.87	18.19 ± 5.60	25.22 ± 6.54	25.68 ± 4.98	23.46 ± 1.15
	<RC>	66.34 ± 3.79	46.11 ± 16.66	58.97 ± 8.80	52.80 ± 5.18	58.19 ± 1.13
Aβ_42−_Cu^2+^/DMPC	<BS>	2.36 ± 1.13	7.94 ± 7.84	4.11 ± 3.80	13.46 ± 4.12	7.02 ± 2.50
	<H>	7.67 ± 4.35	32.10 ± 13.49	16.75 ± 9.94	11.55 ± 4.30	13.39 ± 2.06
	<T>	18.18 ± 9.72	19.69 ± 2.49	27.18 ± 4.97	21.83 ± 4.01	21.08 ± 3.33
	<RC>	71.79 ± 13.15	40.28 ± 16.40	51.95 ± 8.78	53.15 ± 3.42	58.52 ± 3.09

*Note*: Beta sheet, helix, turn, and random coil represented as <BS>, <H>, <T>, and <RC>. The standard error estimated averaging over ensembles obtained at equilibrium.

The average per‐residue secondary structure contents for the peptides with and without^22^ DMPC bilayer are displayed in Figure S1c. The <BS> contents in Gly29‐Val36 and Val39‐Ile41 and the <H > contents in Glu15‐Leu17 observed in Aβ_1–42_‐Cu^2+^/DMPC are completely missing in Aβ_1–42_/DMPC. Nevertheless, a higher tendency of the <H> propensity found in Met35‐Ile41 of Aβ_1–42_/DMPC is transformed into the <BS> by Cu^2+^ binding (see Figure 3). It is noteworthy that R4 (G29‐Ile41) residues of Aβ_1–42_ possessed a higher population of <BS> contents are turned into the <H> conformations in Aβ_1–42_/DMPC. On the other hand, the <H> contents spanning regions R2, R3, and R4 are 12.71%, 6.09%, and 5.59% in the case of Aβ_1–42_ (Figure S1c). Whereas, in the case of Aβ_1–42_/DMPC, the helical formation in the same regions increased to 33.94%, 15.07%, and 20.77%, respectively. Similar trends in Aβ_1–42_‐Cu^2+^ with and without DMPC were observed: R2, R3, and R4 of Aβ_1‐42_‐Cu^2+^ obtained <H> contents are 8.59%, 13.55%, and 6.08% are level up to 32.10%, 16.75%, and 11.43% in Aβ_1–42_‐Cu^2+^/DMPC complex. Interestingly, overall <H> propensity of Aβ_1–42_ (7.63%) enhanced to Aβ_1–42_/DMPC (16.57%); in contrast, <BS> contents decreased from 12.01% of Aβ_1–42_ to 1.71% of Aβ_1–42_/DMPC. The same trend followed, 7.05% of <H> and 11.53% of <BS> found in Aβ_1–42_‐Cu^2+^ are increasing <H> (13.39%) and decreasing <BS> (7.02%) in the case of Aβ_1–42_‐Cu^2+^/DMPC. These pieces of evidence imply that Ca^2+^ ions and DMPC bilayers' interaction promoted higher <H> and lower <BS> in both free and copper bound Aβ_1–42_ peptides. It is worth noting that both Aβ_1–42_ and Aβ_1–42_‐Cu^2+^ monomers undergo a random coil to β‐sheet transition in the aqueous phase and helix transition at the membrane phase (Figure S1c). Fatafta et al.^32^ also observed similar trends for Aβ_1–42_ dimers in the aqueous and membrane phases.

### 3.3 Cu^2+^ binding enriches the stability of Glu22‐Lys28 and Asp23‐Lys28 Salt bridges

**FIGURE 4:**
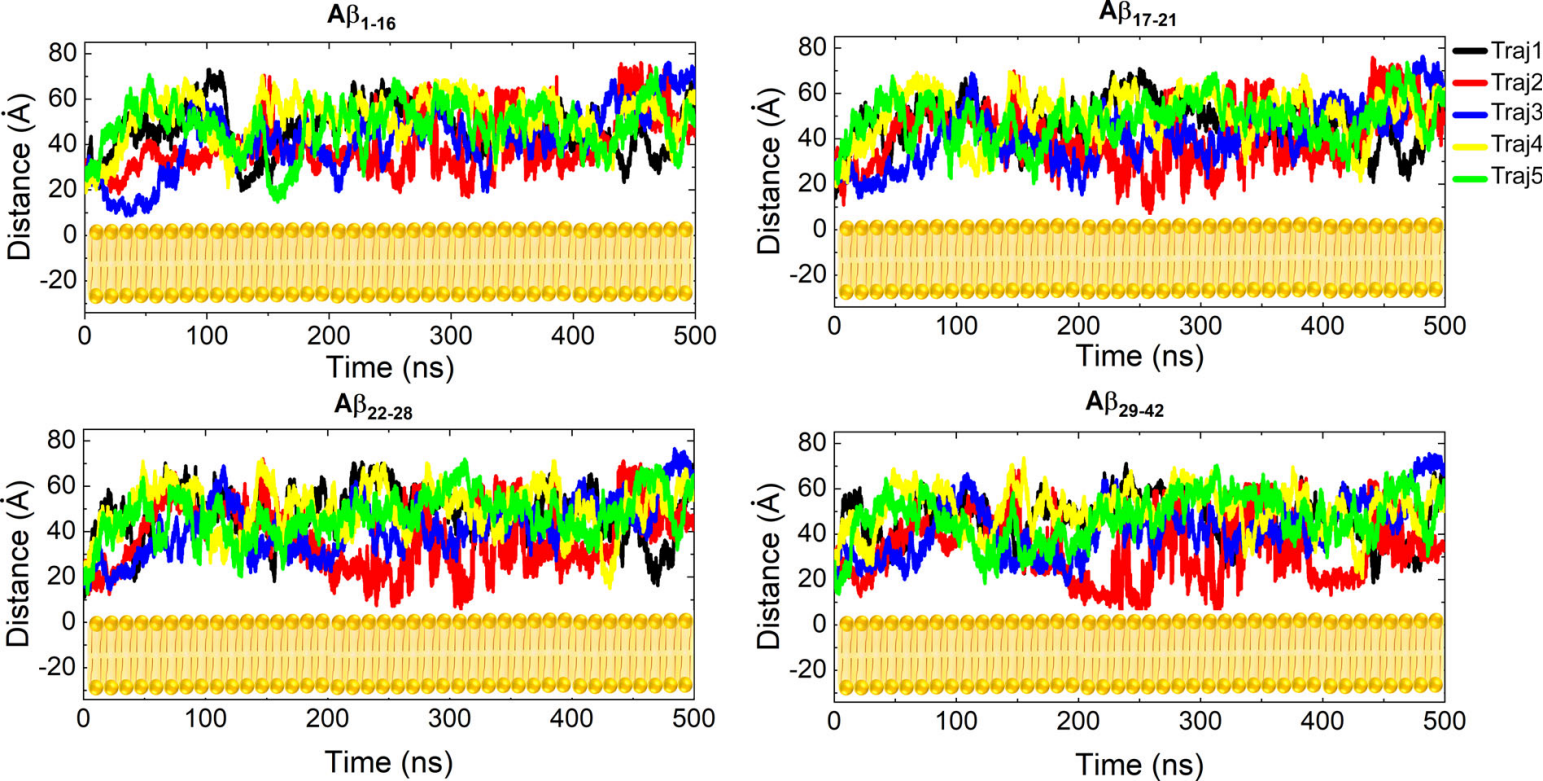
Distance between different region of Aβ_1–42_ peptide and the dimyristoylphosphatidylcholine (DMPC) bilayer (yellow) plotted against time

We verified the effect of Ca^2+^ ions in the relation between Aβ_1–42_/Aβ_1–42_‐Cu^2+^ and the DMPC lipid bilayer by computing the distance between the center of mass (COM) of the peptide and the DMPC bilayer (Figures 4 and S1d) using a distance program in AmberTool 16. We consider contact between two species when the distance between their COM falls less than 8 Å. In the case of Aβ_1–42_/DMPC, trajectory2 has contact with the membranes between 200 and 500 ns (Figure 4). Furthermore, we inspected region‐wise trajectory2 of peptide relation with the membranes and observed R3 and R4 are most frequently participating in the interaction. In contrast, Figure S1d shows that trajectory2 of Aβ_1–42_‐Cu^2+^ contacted the membrane in the first 150 ns. After that, it was away from the membranes. This is interpreted as that Aβ_1–42_, rather than Aβ_1–42_‐Cu^2+^, forms more frequent contacts with the membranes.

**FIGURE 5:**
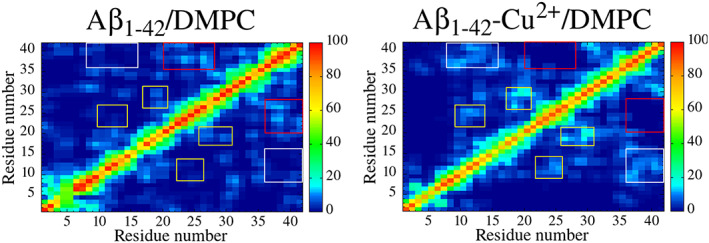
All trajectory ensemble‐averaged intramolecular C_α_ contact map of the Aβ_1–42_ and Aβ_1–42_‐Cu^2+^ peptide

Figure 5 illustrates the prevalence of contacts between two residues for all trajectories of Aβ_1–42_/DMPC and Aβ_1‐42_‐Cu^2+^/DMPC by a percentage of total frames. The contacts have been classified by three types based on a separation between the two residues^66^: (a) long‐range contact is considered when there is a separation greater than 24 residues (highlighted by a white square box in Figure 5), (b) medium‐range contact, separation is 12–23 residues(red square box), and (c) short‐range contacts, separation is between 6 and 11 residues (yellow rectangular). Long‐range contact between residues Ser8‐Lys16 and Val36‐Ala42, medium‐range contacts between residues Gly9‐His13 and Glu22‐Asn27, and short‐range contacts between residues Leu17‐Ala21 and Gly25‐Ala30 were observed in the case of Aβ_1–42_‐Cu^2+^/DMPC, these contacts are nonexistent in the case of Aβ_1–42_/DMPC. The contrast trend followed by Aβ_1–42_/DMPC shows the medium‐range contacts between residues Phe20‐Lys28 and Val36‐Ala42 disappeared in the case of Aβ_1–42_‐Cu^2+^/DMPC.

**FIGURE 6:**
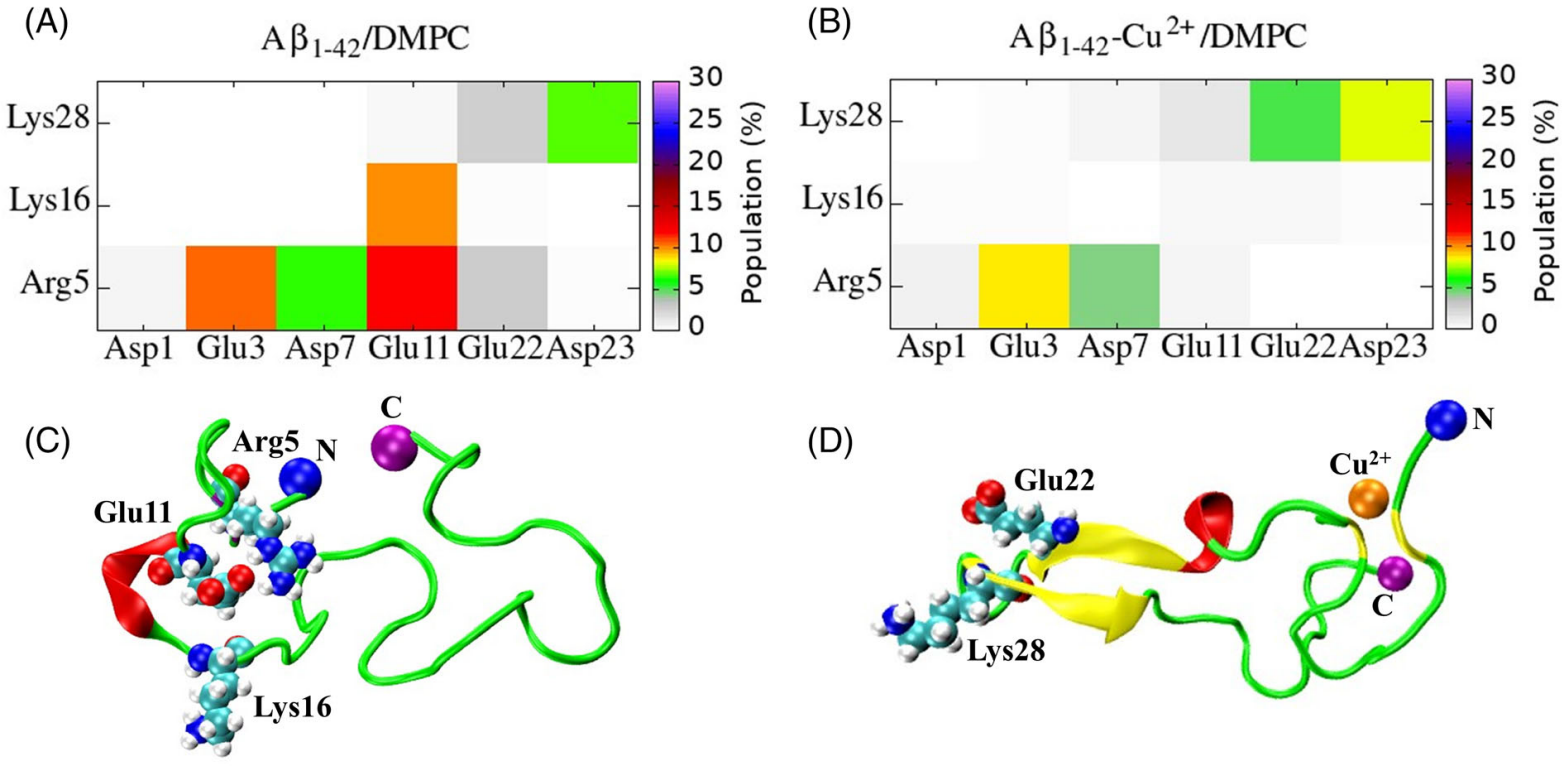
Salt bridge contacts between charged residues for (A) Aβ_1–42_ and (B) Aβ_1–42_‐Cu^2+^. Representative structure of (C) Aβ_1–42_ and (D) Aβ_1–42_‐Cu^2+^ displayed higher population salt‐bridge contact residues are highlighted in van der Waals representation. In the peptide, helix, beta‐sheet and random coil are shown in red, yellow and green, respectively; C, N, O, H and Cu atoms are shown in cyan, blue, red and orange; and N‐ and C‐terminal represented by blue and purple sphere. For the purpose of clarity, membrane was hidden.

The Aβ_1–42_ peptide has three positively charged residues (Arg5, Lys16, and Lys28) and six negatively charged residues (Asp1, Glu3, Asp7, Glu11, Glu22, and Asp23); the total charge is −3; hence, 18 salt‐bridges are possible between the charged residues. The probability of salt bridges contacts of the Aβ_1–42_/DMPC and Aβ_1–42_‐Cu^2+^/DMPC for all trajectories by an average of total frames are shown in Figure 6A,B; the most representative structure of both complexes is displayed in Figure 6C,D. Seven salt‐bridges, Arg5‐Glu3, Arg5‐Glu3, Arg5‐Glu11, Arg5‐Glu22, Glu11‐Lys16, Glu22‐Lys28, and Asp23‐Lys28, were determined in the Aβ_1–42_/DMPC, two of them, Arg5‐Glu11 and Glu11‐Lys16, have the most prevalence with 10%–12% of the population. It is noteworthy that those salt bridges disappeared in the case of Aβ_1–42_‐Cu^2+^/DMPC. Another two salt‐bridges, Glu3‐Arg5 and Arg5‐Asp7, decreased from 10.61% and 6.53% to 8.67% and 4.49% upon Cu^2+^ binding to Aβ_1–42_/DMPC. The results interpreted that the Cu^2+^ binding cause reduced or inhibited the population of five salt bridges, Arg5‐Glu3, Arg5‐Glu3, Arg5‐Glu11, Arg5‐Glu22, and Glu11‐Lys16, in the Aβ_1–42_/DMPC. In other words, Cu^2+^ binding to Aβ_1–42_ decreased the number of salt‐bridges because charged residues side‐chains interact with either Ca^2+^ ions or water molecules.

In general, two salt bridges, Glu22‐Lys28 and Asp23‐Lys28, play an essential role in the β‐hairpin structure found in oligomers and fibrils.^67^ We determined these two‐salt bridge contact populations to be higher in Aβ_1–42_‐Cu^2+^/DMPC compared to Aβ_1–42_/DMPC are playing a pivotal role in stabilizing turn conformation at the loop region. In particular, in the case of Aβ_1–42_‐Cu^2+^/DMPC, Glu22‐Lys28 salt bridge population has two folders higher than another case, and the main feature of this salt bridge is to trigger β‐hairpin conformation at Leu17‐Glu33 residues by the stabilized turn structure of Glu22‐Lys28 connected between two beta‐sheet appeared at residue Leu17‐Ala21 and Gly29‐Gly33 (Figure 6D). Whereas in the case of Aβ_1–42_/DMPC, unstructured conformation exists in the residues mentioned earlier (Figure 6D) since the weaker population of Glu22‐Lys28 salt‐bridge.

Next, we compared these results with our previous investigation of the same peptide without Ca^2+^ ions and DMPC bilayer.^22^ Notably, we found that Cu^2+^ binding to Aβ_1–42_ can decrease three salt bridges (Arg5‐Glu3, Arg5‐Glu11, and Glu11‐Lys16) population and increase two salt bridge (Glu22‐Lys28 and Asp23‐Lys28) population at both the presence and absence of Ca^2+^ and DMPC bilayer. The DMPC and Ca^2+^ ions presence can mediate the disappearance of seven salt bridges (Asp1‐Arg5, Asp1‐Lys16, Asp1‐Lys28, Glu3‐Lys16, Glu3‐Lys28, Arg5‐Asp23, Asp7‐Lys16, and Asp7‐Lys28) in the Aβ_1–‐42_. On the other hand, four salt bridges (Asp1‐Arg5, Asp7‐Lys16, Asp7‐Lys28, and Arg5‐Asp23) were destroyed in Aβ_1‐42_‐Cu^2+^ by the presence of Ca^2+^ and DMPC bilayers.

**FIGURE 7:**
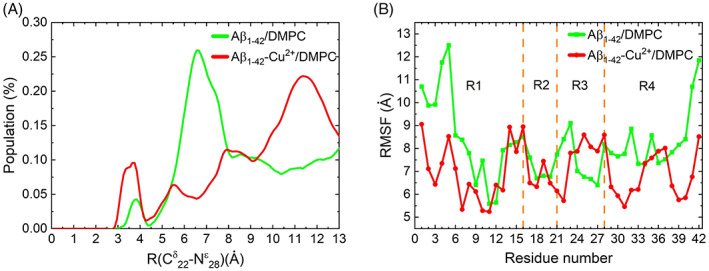
(A) Distribution of salt‐bridge distance between atoms C^δ^_22_ (Glu22) and N^ε^_28_ (Lys28); (B) root mean square fluctuation (RMSF) data (Å) based on individual residues, dotted lines represented the different regions: R1 (residues 1–16), R2 (residue 17–21), R3 (residues 22–28) and R4 (residues 29–42).

We carefully inspected the contact variation between Glu22 and Lys28 residues to the distance between C^δ^ of Glu22 and N^ε^ of Lys28 (Figure 7A). A salt bridge is formed if the distance between the two representative atoms falls within 5 Å. In the case of Aβ_1–42_‐Cu^2+^/DMPC, the Glu22‐Lys28 salt bridge is formed at a distance range of 3.5–4.5 Å. In contrast, no salt bridge is observed in the case of Aβ_1‐42_/DMPC, but weaker contacts are formed at a distance between 5.0 and 7.0 Å. This result confirms that Cu^2+^ binding could increase Glu22‐Lys28 salt bridge stability in Aβ_1–42_/DMPC.

Figure 7B displays the averaged root‐mean‐square fluctuation (RMSF) of the Aβ_1–42_ peptide individual residues and describes the residues' mobility. In the case of Aβ_1–42_‐Cu^2+^/DMPC as compared with Aβ_1–42_/DMPC, most residues in R1, R2, and R4 residues (except Val12, His14, Lys16, Phe19, Gly36, and Gly37) showed low RMSF values, and those in R3 (except Asp23) showed high RMSF values. We found that the C=O group of Asp23, Glu22, and Val24 formed hydrogen bonds to the N—H group of Ser26, Val24, and Asn27 residue, respectively. Those hydrogen bonds that occurred between *i* and *i* + 2 or *i* + 3 amino acid possessed a higher population relative to the peptide Cu^2+^ bound (Figure S1c), maintaining a helical structure at residues Asp23‐Lys28, which has less mobility as compared to the turn or random coil structure of the identical residues generated by Cu^2+^ binding. This information suggests that Cu^2+^ binding to Aβ_1–42_/DMPC can reduce the mobility of the R1, R2, and R4 and increase the flexibility of R3 (Figure 7B). In contrast, the opposite trend was followed by Aβ_1–42_ upon binding Cu^2+^ in the absence of DMPC and Ca^2+^ ions.^22^

### 3.4 Cu2+ binding triggered the Aβ_1–42_ monomer exerts higher hydrophobicity character

**FIGURE 8:**
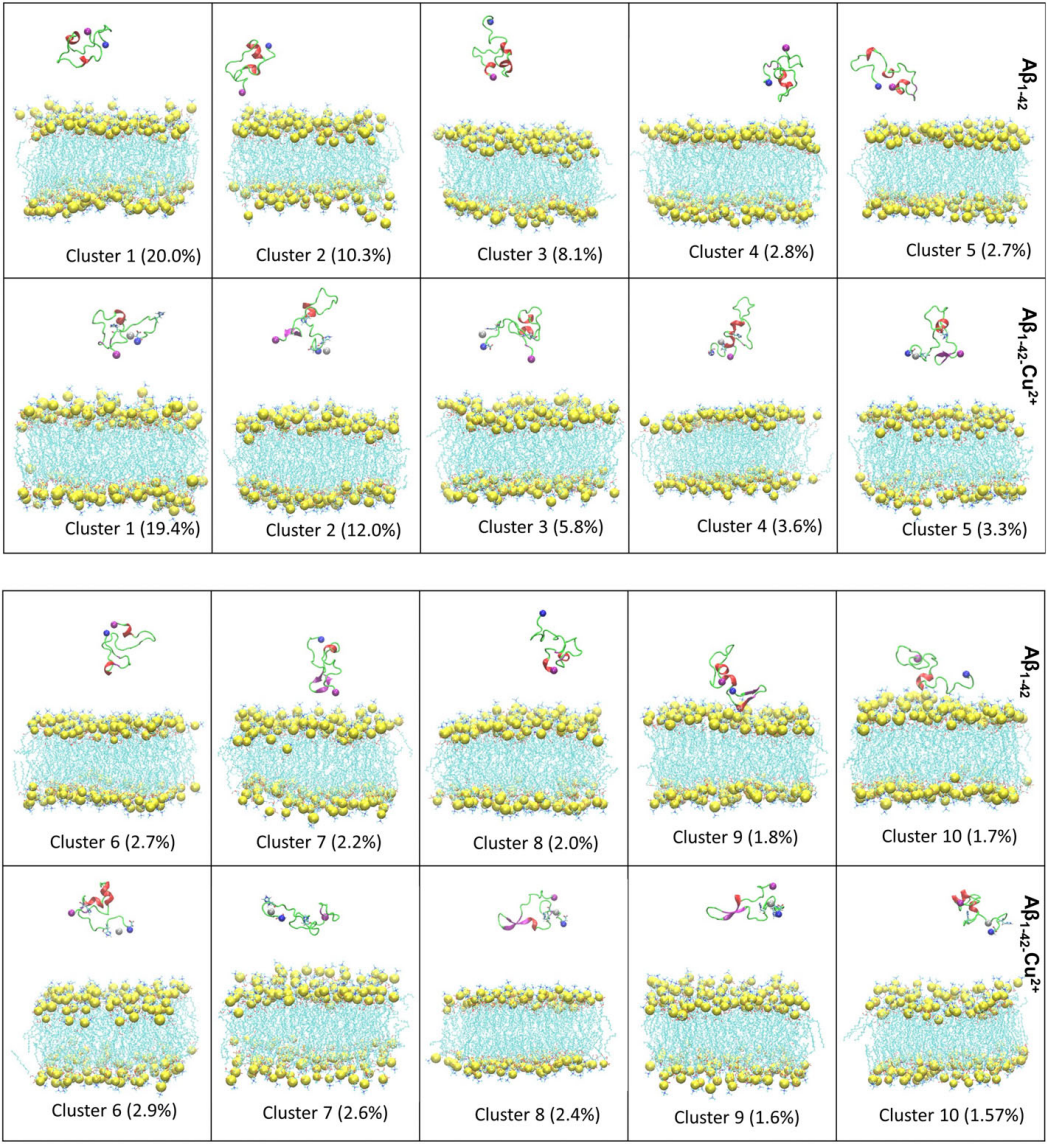
The 10 topmost clusters for Aβ_1–42_ and Aβ_1–42_‐Cu^2+^ are displayed, and the corresponding population is given in percentage. The helix, beta‐sheet, and random coil are highlighted by red, purple, and green, respectively; N‐ and C‐terminal by blue and purple sphere; and Cu^2+^ is represented by a silver sphere. Cu^2+^ binding residues of Asp1, His6, and His13 are shown in licorice representation.

A K‐clustering analysis was performed on the Aβ_1–42_/Aβ_1–42_‐Cu^2+^ peptides using the CPPTRAJ tool based on RMSD with a 4.0 Å cut‐off distance; the results are shown in Figure S2. The analysis of all trajectories shows that Aβ_1–42_/DMPC has 170 clusters, while Aβ_1–42_‐Cu^2+^/DMPC has 190 clusters. The top 10 clusters are shown in Figure 8 and confirmed Cu^2+^ binding could reduce cross‐talk between N‐terminal and C‐terminal.

**FIGURE 9:**
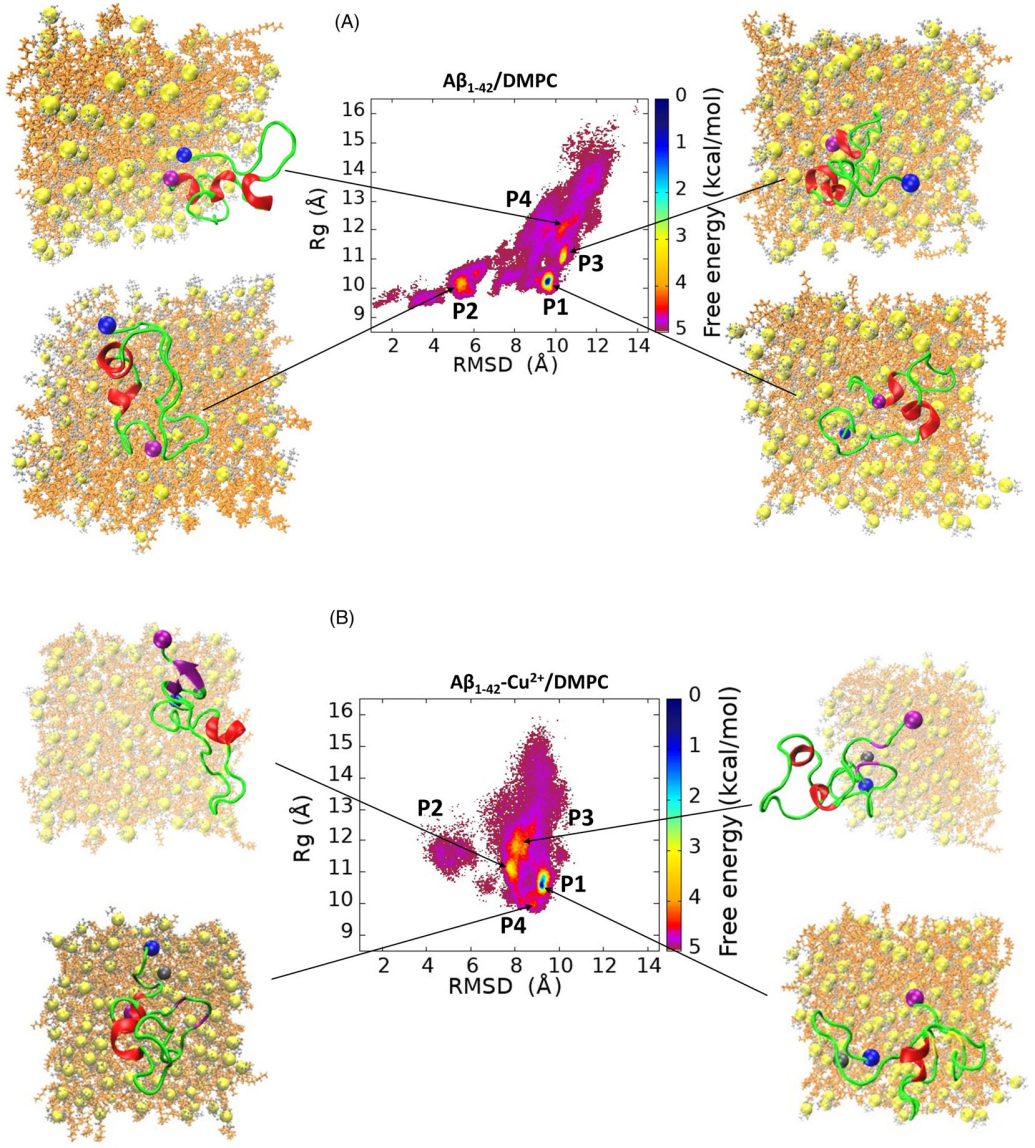
Free energy surface map (in kcal/mol) of (A) Aβ_1–42_ and (B) Aβ_1–42_‐Cu^2+^ peptide as a function of radius of gyration (Rg) and backbone RMSD. Results were based on all conformers of the five trajectories. The free energy surface containing four minimum energy basins called P1, P2, P3, and P4; representative structure in each minimum‐energy basin displayed; and their population are 20.00%, 10.26%, 8.15%, and 2.77% for Aβ_1‐42_ and 19.36%, 11.97%, 5.77%, and 3.65% for Aβ_1–42_‐Cu^2+^. The peptide is shown in cartoon representation; C‐, N‐terminal and Cu in the blue, purple and gray sphere; and helix, beta‐sheet and random coil in red, purple and green, respectively

The FES plot of the Cα RMSD versus the radius of gyration was computed using Equation (3), as shown in Figure 9, and the structural propensities of the most representative conformers are tabulated in Table 3. In the FES of Aβ_1–42_/DMPC (Figure 9A), four local minimum states, P1, P2, P3, and P4, are located with coordinates (RMSD, Rg) at (9.66,10.22), (5.57,10.18), (10.25,11.02) and (10.32,12.10), representing 31.18% of the total. Values of RMSD and Rg fluctuated in the range of 5.57–10.32 Å and 10.18–12.10 Å, respectively. On the other hand, the FES of Aβ_1–42_‐Cu^2+^/DMPC (Figure 9B) was characterized by four local minimum states located at (9.28, 10.59), (7.87, 11.06), (8.09, 11.89), and (9.05, 9.99), representing 40.75% of the total. Values of RMSD and Rg fluctuated in the range of 7.87 Å–9.28 Å and 9.99–11.89 Å, respectively. The results imply that Aβ_1–42_/DMPC exhibits a lower number of clusters, a higher population of P1–P4 states, and larger RMSD and Rg variation than the Aβ_1–42_‐Cu^2+^/DMPC case. It provided evidence of the Cu^2+^ binding reduced the Aβ_1–42_ peptide mobility. Figure 7B confirms R1 (residue Asp1‐Tyr10) and R4 (Gly29‐Gly33, Met35, and Gly38‐Ala42) contribute the most to the Aβ_1–42_ peptide flexibility presenting larger RMSF compared to the Aβ_1–42_‐Cu^2+^ peptide. The Cu^2+^ binding induced significantly structural diversity in Aβ_1–42_, which causes conformers larger spreading at each minimum energy basin (Figure 9A) than the FES of Aβ_1–42_ (Figure 9B). These results are consistent with the REMD simulations^68^ where it is observed that Cu^2+^ binding to Aβ can generate a polymorphic state through more segregated conformations.

**TABLE 3:** The structural propensity of the most representative conformers in each minimum energy basin identified from the free energy surface in Figure 9

System	State	P (%)	RMSD (Å)	Rg (Å)	HB	SASA (Å^2^)	<BS>	<H>	<T>	<RC>
Aβ_1–42_	P1	20.00	9.66	10.22	16	3262	19.04	0.00	23.80	57.14
	P2	10.26	5.57	10.18	17	3014	26.19	0.00	16.66	57.14
	P3	8.15	10.25	11.02	16	3083	28.57	0.00	11.90	59.76
	P4	2.77	10.32	12.10	17	3579	23.80	4.76	16.66	54.76
Aβ_1–42_Cu^2+^	P1	19.36	9.28	10.59	15	3322	16.66	4.76	9.52	69.04
	P2	11.97	7.87	11.06	21	3290	9.52	4.76	30.95	54.76
	P3	5.77	8.09	11.89	16	3321	19.04	4.76	23.80	52.38
	P4	3.65	9.05	9.99	16	3670	9.52	4.76	38.09	47.61

Abbreviations: RMSD, root‐mean‐square deviation; Rg, radius of gyration; SASA, solvent‐accessible surface.

To evaluate the effect of Cu^2+^ binding to Aβ_1–42_, we determined the hydrophobicity and hydrophilicity character of the peptide by performing SASA analysis. The SASA value of the P1–P4 states of free and Cu^2+^ bound peptides is tabulated in Table 3. The average SASA of P1–P4 of Aβ_1–42_ and Aβ_1–42_‐Cu^2+^ are 3244 and 3400 Å^2^, respectively, indicating that the free peptide has a lower hydrophobicity than in the copper bound complex. In addition, the SASA value per residue differences between the four FES local minima of Aβ_1–42_ and Aβ_1–42_‐Cu^2+^ as shown in Figures S3 and S4. The positive and negative values of SASA differences in Figure S4 represent hydrophobicity and hydrophilicity character, respectively. We found Cu^2+^ binding can increase the SASA value of the Aβ_1–42_ by 60, 276, and 242 Å^2^ in the P1, P2, and P3, respectively (Table 3).The R1 and R4 have significant contributions to enhancing the hydrophobicity in the P1–P3 states of Aβ_1–42_‐Cu^2+^ (Figure S4) through three significant events (a) Cu^2+^ binding residues of Asp1, His6, and His13 or His14 in R1 are more exposed to the waters molecules compelling neighboring residues to enhance their hydrophobicity, (b) significant secondary structure changes at helical contents of R4 transforms it into a β‐sheet structure that exhibits higher hydrophobicity and (c) increase in the number of hydrogen bonds ranges from 16–17 to 15–21 between amino acids in the full‐length of Aβ_1–42_.

Experimental studies^53^, ^54^, ^69^, ^70^ have demonstrated that helix contents in the C‐terminal of Aβ favor interaction with the lipid bilayer. This observation is consistent with the present simulation of helical structure formation at C‐terminal, which involves interaction with the lipid bilayer by van der Waals in the case of the free peptide. In contrast, Asp1, His6, and His13/His14 involved in Cu^2+^ metal coordination at N‐terminal facilitate a beta‐sheet structure at the C‐terminal, leading to decreased mobility and increased hydrophobicity (higher SASA) of the Aβ_1–42_ peptide, as a result preventing the interaction with the membranes. Subsequently, another significant change was noted: the salt bridge formation at Glu22‐Lys28 promoted a beta‐hairpin conformation at Leu17‐Glu33 residues of Aβ_1–42_ due to the Cu^2+^ coordination (Figure 3).

### 3.5 Ca^2+^ inhibits Aβ monomers' penetration into the membrane

We examined the distribution of amino acids *i* along the *z*‐axis to the bilayer P (*z*, *i*) for each residue in the N‐terminal, central‐hydrophobic, loop, and C‐terminal regions (Figure S5). In the N‐terminal region, the five residues, Arg5, His6, Asp7, Ser8, and Gly9, have a maximum in the range of 13–16 Å, which is less than the average position of the center of masses of phosphorous atoms Zp = ~17 Å, indicating that these five residues are inserted into the bilayer. In the Central‐hydrophobic and Loop regions, residues Leu17‐Ala21 and Glu22‐Lys28 have a maximum at distances ca. *Z* = 22 Å (Z > Zp), indicating that these residues participate in the bilayer surface interactions. In the C‐terminal, Val39, Gly38, and Val40 have peaks near Zp, while residues Gly29, Ala30 and Gly37 have a maximum at about 19 Å, meaning that the former residues are inserted in the bilayer and latter residues form strong bilayer surface contacts. These results confirmed that N‐ and C‐terminal residues are involved in the mechanism of bilayer insertion and that the central and those residues at the loop region form contacts with the lipid bilayer.

**FIGURE 10:**
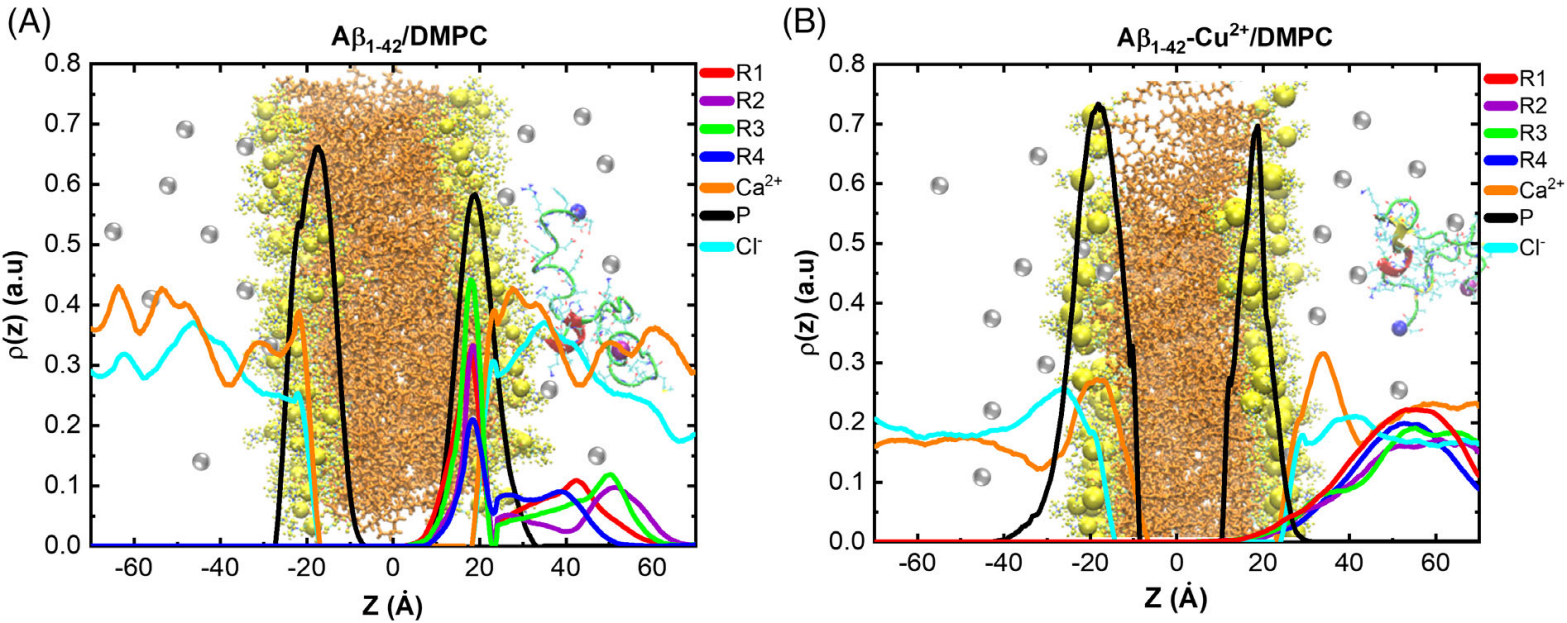
Density profiles for different atomic groups along with the *z*‐axis coordination of bilayer. The density mass of each group is divided by the number of atoms in each group. R1 contains residues Asp1‐Lys16, R2 Leu17‐Ala21, R3 Glu22‐Lys28, and R4 Gly29‐Ala42. Membrane‐peptide complex displayed in the background, where calcium ions showed in silver color, phosphorous atoms in yellow sphere, and peptide in cartoon representation; for clarity, water and Cl^−^ ions are not shown.

We also inspected the mechanism of peptide insertion by plotting the density profile for peptide, lipid, and ions versus the z‐axis of the lipid bilayer (Figure 10). The total density of the simulation system, shown in Table 1, is partitioned into a peptide, CaCl_2_, DMPC lipid bilayer, and water. The density maps of different atomic sets such as R1, R2, R3, and R4 of the peptide, Phosphorous atoms of DMPC, Ca^2+^, and Cl^−^ ions were obtained by taking as reference the center of the bilayer thickness (Zp = 0). The density of each component is divided by the number of atoms in each set. The higher density of phosphorous atoms at Zp ~ −17 Å and Zp ~ +17 Å. The Ca^2+^ and Cl^−^ ions density is zero between the ~−17 and ~+17 Å in the case of both Aβ_1–42_/DMPC and Aβ_1–42_‐Cu^2+^/DMPC complexes. The distances between the density of the upper layer and the lower layer of the phosphorous atoms are called bilayer thickness. The estimated bilayer thickness ~34 Å is consistent with experimental data^71^ of lipid bilayer thickness.

The highest density of the Aβ_1–42_ R1–R4 regions was found in the range of 17–20 Å (Figure 10A), suggesting the peptide is closer to the phosphorous atoms at the upper membrane layer. Whereas in Aβ_1–42_‐Cu^2+^/DMPC (Figure 10B), the density of the R1–R4 regions was found beyond 40 Å, suggesting that the peptide is distant from the lipid bilayers. Nevertheless, a significantly higher density of Ca^2+^ ions appeared between lipid bilayers and the peptide. As a result, the Ca^2+^ inhibited the peptide‐membrane contact in the case of Aβ_1–42_‐Cu^2+^/DMPC. These results suggest that: (a) Aβ_1–42_ charges (−3) attract the positively (+2) charged Ca^2+^ ions which mediate the peptide‐membrane interactions; (b) Cu^2+^ binding reduced total charges of Aβ_1‐42_ from −3 to −1, and the presence of Ca^2+^ ions facilitate the reduced attraction of the peptide by the membrane, which prevents the complete contact with the membranes. Therefore, Ca^2+^ ions mediated Aβ_1–42_ interactions with the membrane were prevented by Cu^2+^ binding to the peptide.

### 3.6 Ca^2+^ impedes the Aβ_**1–**42_‐membrane binding affinity

**FIGURE 11:**
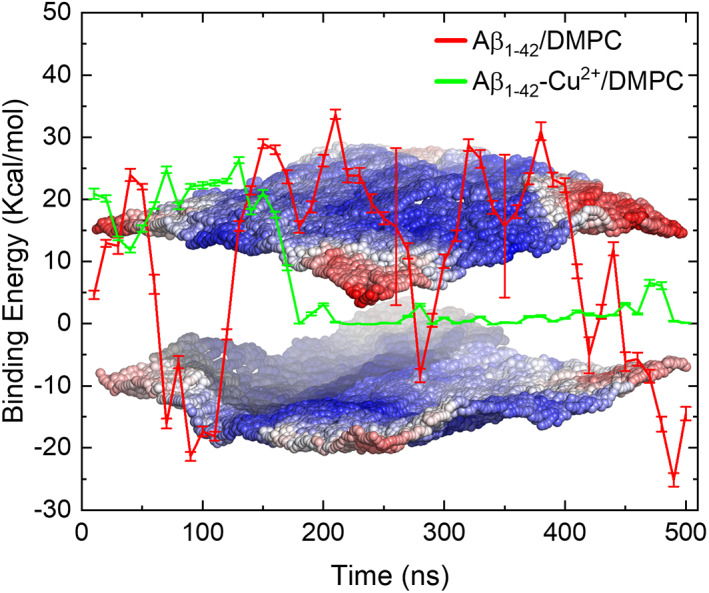
MM‐GBSA binding free energies at every averaged 10 ns windows ± standard error over 500 ns simulation for Aβ_1–42_/DMPC and Aβ_1–42_‐Cu^2+^/DMPC complexes. The background figure shows the membrane thickness of the latter complexes using the g_lomepro tool, the thickness increases as a red‐white‐blue color gradient. The result revealed that Aβ_1–42_‐Cu^2+^ did not perturb the membrane.

Figures 4 and S1 show that the trajectory of replica 2 places Aβ peptides in close contact with the lipid bilayer; therefore, we explored the binding energy profile for interactions between the peptide and the P0_4_ group of the DMPC bilayer using the MM‐GBSA method. The binding free energies for every 10 ns averaged windows over the 500 ns of simulation are displayed in Figure 11. The negative and positive values represent increasing and decreasing the binding energies. We noted that the higher binding energies persist in the range of 1–140 ns and 400–500 ns in the case of Aβ_1–42_/DMPC, whereas in the case of Aβ_1–42_‐Cu^2+^ complex the positive binding energy values suggest that favorable interactions are not formed.

Van der Waals (Δ*E*_vdw_), electrostatic (Δ*E*_elec_), and binding free energy (Δ*G*_bind_) between peptide and membrane are tabulated in Table 4. In the case of Aβ_1–42_/DMPC, Δ*E*_vdw_ value is about −8.0 kcal/mol lower than the Δ*E*_elec_ value, implying that van der Waals energy is the dominant contribution to the Aβ_1–42_‐DMPC bilayer binding energy (Δ*G*_bind_ = −5.93 kcal/mol). On the other hand, upon Cu^2+^ binding to the Aβ_1–42_ peptide both Δ*E*_vdw_ and Δ*E*_elec_ values are higher than those found for the unbound Aβ_1–42_. Notably, the total peptide‐membrane binding energy (Δ*G*_bind_) is repulsive upon Cu^2+^ binding; as a result, Cu^2+^ binding can reduce or inhibit the peptide‐membrane contact.

**TABLE 4:** MMGBSA binding free energy between peptide and membrane (kcal/mol) ± standard deviation for the last 100 ns of trajectory 2

	Aβ_1–42_/DMPC	Aβ_1‐42_‐Cu^2+^/DMPC
Δ*E*_vdw_	−36.06 ± 3.37	−0.63 ± 0.72
Δ*E*_elec_	−28.38 ± 14.68	−20.51 ± 10.93
Δ*G*_bind_	−5.93 ± 5.60	0.90 ± 0.97

Abbreviation: DMPC, dimyristoylphosphatidylcholine.

We observed that the Δ*G*_bind_ energy between Aβ_1–42_ monomer and zwitterionic DMPC bilayer is −5.93 kcal/mol in the presence of higher concentration Ca^2+^ ions. Interestingly, David and Berkowitz^72^, ^73^ have determined the Δ*G*_bind_ energy between Aβ_1–42_ monomer and zwitterionic DPPC bilayer as −14.42 kcal/mol in the absence of Ca^2+^ ions and identified the monomer highly attracted by the surface of the lipid membrane. It drives Aβ_1–42_ peptide spreading on the membranes leading to aggregation by peptide–peptide interactions. Our findings strongly suggest that Ca^2+^ ions can decrease binding affinity between the Aβ_1–42_ monomer and the zwitterionic membranes by increasing ~2.5 times of Δ*G*_bind_ values (from −14.42 to −5.93 kcal/mol). In other words, Ca^2+^ ions can delay the Aβ_1–42_ monomer form oligomer structures due to decreasing binding affinity between the peptide and membranes.

**FIGURE 12:**
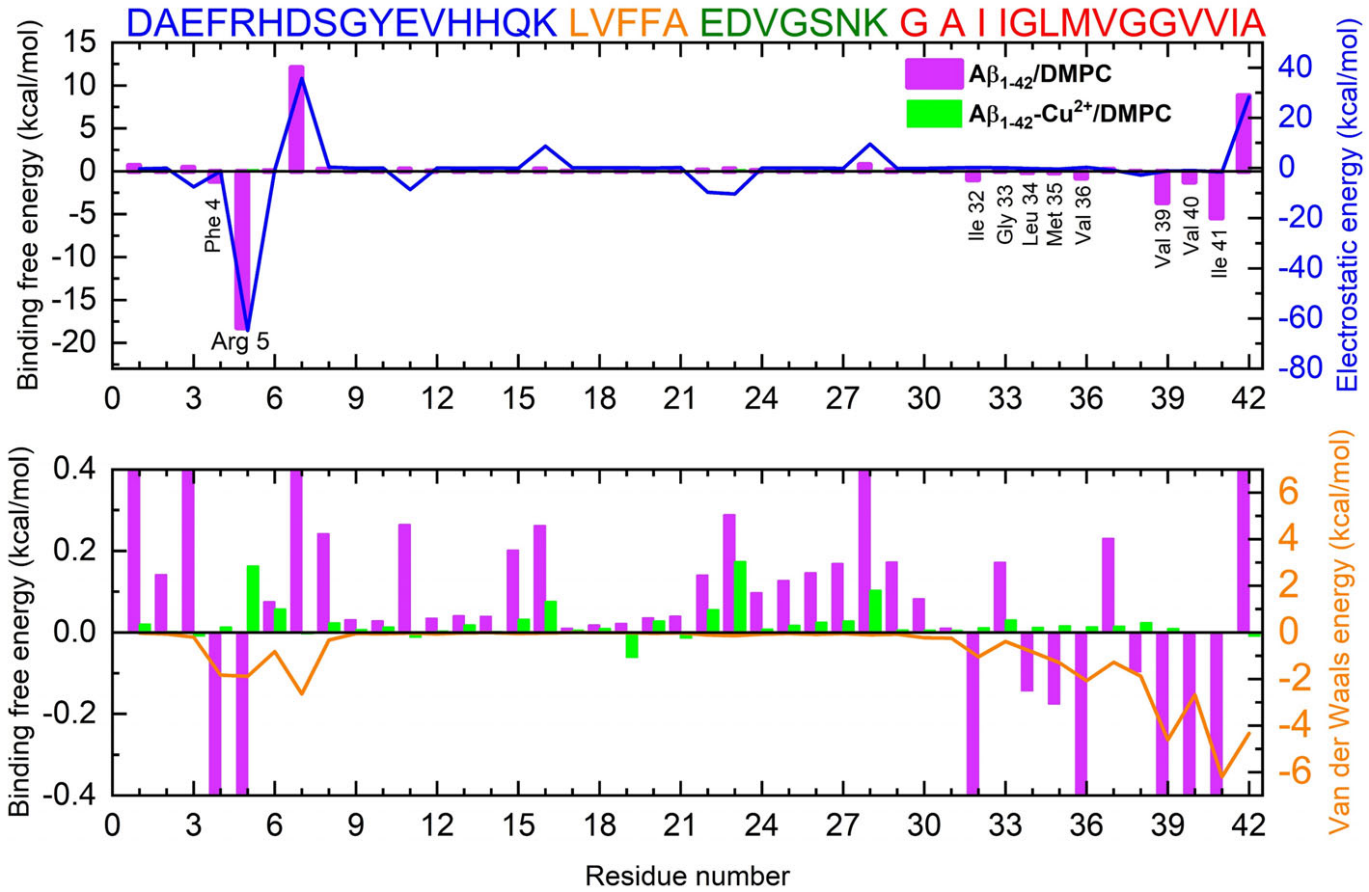
MM‐GBSA method was used to calculate the residue‐wise contributing van der Waals energy, electrostatic energy, and the binding free energy between peptide (Aβ_1–42_ and Aβ_1–42_‐Cu^2+^) and DMPC bilayer. The bottom figure is a magnified version of top one

We deeply examined the residue contribution to the binding energy in the interaction of the PO_4_ group of DMPC bilayer (see Figure 12). We observed two significant changes in Aβ_1–42_/DMPC. First, Arg5 residue has the lowest binding energy (−18.23 kcal/mol), due mainly to a strong electrostatic interaction (−64.75 kcal/mol), which enhances the Arg5–PO_4_ interactions. Second, C‐terminal residues, Ile32, Leu34‐Val36 and Gly38‐Ile41, have received binding energy −12.48 kcal/mol, whose most significant contribution comes from van der Waal interactions (sum of ΔE_vdw_ = −20.64 kcal/mol) rather than electrostatic interactions (sum of ΔE_elec_ = −6.87 kcal/mol). These observations suggest that, in the case of Aβ_1–42_/DMPC peptide, N‐terminal and C‐terminal residues actively participated in the lipid bilayer interaction through electrostatic and van der Waals interactions; however, in the case of Aβ_1–42_/DMPC peptide, those interactions are nonexistence.

## 4 DISCUSSIONS

It is known that the interaction of Aβ peptides with the neuronal membrane leads to a significant contribution to cognitive deficits associated with AD. Several experimental efforts have been addressed to exploring the interaction between Aβ oligomers and the membranes,^74^ for instance, (1) surface plasma resonance spectroscopy^75^ has shown that the interaction between Aβ and membrane was weaker for the POPC bilayer compared to the POPG bilayer, (2) quasi‐elastic neutron scattering spectroscopy^76^, ^77^ studies reported that peptide–peptide interaction dictates the Aβ_25–35_ and Aβ_22–40_ peptide diffusion into the mixed DMPC/DMPS bilayer, destabilized membrane stability and increased membrane fluidity, and (3) small‐angle neutron scattering and neutron diffraction studies^78^ show that Aβ_1–40_ peptides were barely bound to anionic DMPG bilayer, resulting in altering membrane dynamics, and it does not deeply penetrate the bilayers. Single‐molecule microscopy^79^ studies confirmed that Aβ_1–42_ peptides developed a higher aggregation than Aβ_1–40_ peptides in hippocampal neuronal rats; Aβ_1–42_ monomers are associated with the membrane and exert dynamics characters highly; trimers and oligomers are immobile on the membrane. The less immobilized monomer has a lower propensity to insert membrane.

Although experimental approaches have not determined the direct peptide‐membrane interaction on the monomeric level, MD simulations are a suitable tool for this system. For instance, David and Berkowitz^73^ have inspected the dimer formation of the Aβ_1–42_ peptides on zwitterionic DPPC and anionic DOPS bilayers using MD simulations. They found that the DOPS bilayer is favorable for Aβ_1–42_ dimerization by augmenting peptide–peptide binding, the opposite trend followed by DPPC bilayer, which enhances the interaction between Aβ_1–42_ peptide and membranes. The pieces of evidence imply that the DOPS bilayer mediated aggregation‐prone structure. Ngo et al.^80^ have documented that van der Waals interactions rather than electrostatic interactions are dominant in elevating the binding between Aβ_11–40_ trimer and DPPC bilayer, resulting in the trimer quickly diffusion into the bilayers.

On the other hand, the Aβ_1–42_ peptides are arranged in parallel orientation with the DPPC bilayers by forming strong electrostatic attraction of anionic N‐terminus to the zwitterionic head group. Nevertheless, the same peptide adopted perpendicular arrangement on the anionic DOPS bilayer membrane due to stronger electrostatic repulsion between anionic N‐terminus and the anionic head group, drives enhancing peptide–peptide interaction for oligomer formation.^72^ In addition, on the DPPC bilayer, the Asp23‐Lys28 salt bridge stabilized the β‐hairpin conformation in Aβ_1‐42_ peptide, which are reduced on the DOPS bilayer through breaking the salt bridge where Asp23 has a stronger binding with the headgroup of the membrane.

Recently, Huy Pham et al.^40^ have found that Mg^2+^ binds phospholipids head groups to weaken the membranes' polarization and destabilize the DMPC bilayer. The monomeric Aβ_1–42_ peptides can prevent membrane destabilization by blocking the contact between divalent metal ions and the membranes. Lockhart and Klimov^9^ have performed MD and REMD simulations to probe the impact of Aβ_1–40_ binding on anionic DMPS bilayer and their outcomes compared to the peptide bind zwitterionic DMPC bilayer. A high population of alpha‐helix in the C‐terminal, decreased intrapeptide interaction, higher electrostatic interaction between charged amino acids and charged lipid groups, and extensive membrane integrity dissociation were observed by Aβ binding to DMPS rather than DMPC. Smith et al.^14^ have monitored Aβ_23–35_ aggregation within DMPC bilayer using REMD simulation. They found that (a) Aβ_23–35_ peptides form rapidly into dimers structure, and (b) monomeric peptides contributed to deeper penetration in the membrane causing big pore formation in the membranes in which uncontrollable calcium ions transport occurred leading to extensive neuronal damage.

Our previous review^5^ extensively discussed the efforts of several groups on the interaction of Aβ monomer, dimer, trimer, and oligomer with the different lipid membrane. Even though the researchers identified the link between calcium dyshomeostasis, Aβ‐membrane interactions, and toxicity could cause neuronal damage,^5^ the mutual interference between Aβ_1–42_/Aβ_1–42_‐Cu^2+^ and DMPC bilayer in the presence of Ca^2+^ ions has not been reported in the literature, which is a necessary detail to unveil the Aβ_1–42_‐Cu^2+^ toxicity properties. To examine the underlying mechanism, Lockhart and Klimov^50^ have inspected the Aβ_10–40_ monomer binding character with the DMPC bilayer in the presence of Ca^2+^ ions using REMD simulation. They reported that Ca^2+^ ions mediate the monomer penetrating the bilayer by the electrostatic interactions between charged residues of the monomer and polar lipid groups. Smith et al.^81^ found that Aβ_25–35_ and Aβ_10–40_ binding with the DMPC bilayer showed different behavior, primary inducing minor depletion in the lipid bilayer and C‐terminal of the latter diffusion into the bilayer deeply, which causes extensive damage to the membrane.

On the other hand, the interaction between Aβ and Cu^2+^ on the Aβ conformations have been characterized in several simulation studies,^5^, ^19^, ^31^ which hypothesized that Cu^2+^ involves higher binding affinity with Aβ, allowing: (a) catalysts formation for the production of reactive oxygen species, activating dioxygen molecules and yielding oxidation pathways, and (b) enhancement of the proportions of beta‐sheet and alpha‐helical structures in Aβ peptide; triggering amorphous aggregation by preventing Aβ from transforming into fibril structures. Still, exploring the mutual relationship between divalent cations and zwitterionic membranes is the most challenging research to address neuronal cell death. Indeed, a recent investigation^82^ explored the interaction mechanism of divalent (Ca^2+^ and Na^+^) with the membranes and found Ca^2+^ binding to the membrane is stronger than Na^+^.

To determine the specific residues of Aβ_1–42_ peptide participated in the interaction with the membrane is an ongoing debate in understanding oligomerization mechanism; thus, we have characterized the connections between the Aβ and DMPC bilayers by exploring the distance between the center of mass (COM) of the peptide and the COM of the bilayers. Figures 4 and S1d displayed the distance profile between Aβ_1–42_‐DMPC, Aβ_1–16_‐DMPC, Aβ_17–21_‐DMPC, Aβ_22–28_‐DMPC, and Aβ_29–42_‐DMPC for 500 ns of each of the five trajectories. The distance of Aβ_17–21_ and Aβ_22–28_ regions of the trajectory2 to the DMPC bilayers lies below 6.5 Å, indicating these regions have participated in the interaction of the bilayer compared to other cases, those are persisting the longer distance (>10 Å). Recent studies demonstrated that the robust binding of the Aβ_10–40_ and Aβ_1–42_ to the DMPC bilayer drives the peptide to penetrate the bilayers.^40^, ^83^ It is noteworthy that our present results interpreted that the presence of calcium ions significantly reduced (inhibited) the contact between the Aβ_1–42_ (Aβ_1–42_‐Cu^2+^) peptide and DMPC bilayers (see Figure 2). Interestingly, these observations corroborated with single‐molecular microscopy^84^, ^85^ experiments study that interactions between Aβ peptides triggered larger aggregates and impeded the Aβ association with the membranes at the hippocampal neurons of the rat.

We discuss structural properties; in the case of Aβ_1–42_, four helical contents appeared at Phe19‐Ala21, Val24‐Asn27, Ile32‐Met35, and Gly38‐Ile41 connected by three turn structures found at Glu22‐Asp23, Lys28‐Ile31, and Val36‐Gly37 (Figure 3). In contrast, two β‐hairpin conformations were noted in Aβ_1‐42_‐Cu^2+^, the first β‐hairpin was found at Phe20‐Ala30 by the turn of Asp23‐Asp27 connected between two β‐sheet at Phe20‐Glu22 and Lys28‐Ala30 (Figure 3). Two significant contributions stabilized this hairpin: (a) hydrophobic interactions between Ala21 and Gly29, and Phe20 and Ala30 (Figure 5) via hydrogen bond most frequently occur (Figure S6), and (b) salt‐bridge formation population between Glu22 and Lys28 (Figure 7). The second β‐hairpin appeared at Leu34‐Ile41 with a turn at Val36‐Gly37 and two β‐sheet at Leu34‐Met35 and Gly38‐Ile41. It has been stabilized by the number of hydrophobic contacts between the β‐sheets. The Cu^2+^ binding significant contribution to enhancing β‐sheet formation in Aβ_1–42_ led to higher hydrophobicity characters and less mobility, which may trigger to faster amorphous aggregation pathway. In addition, salt bridge (Figure 6) and cluster (Figure 8) analyses revealed that Cu^2+^ binding to Aβ_1–42_ decreased the number of salt‐bridges, implying no stronger cross‐talk between N‐terminal and C‐terminal because charged residues side‐chains interact with either Ca^2+^ ions or water molecules.

We discussed earlier studies that confirmed the truncated and full‐length of the Aβ_1–42_ peptide diffusion into the membranes. Pham et al.^40^ predicted insertion of the Aβ_1–42_ and Aβ_1–42_‐Cu^2+^ into DMPC bilayer without Ca^2+^ ions. Yu and Zheng^86^ revealed that interactions between charged residues of Aβ_1–42_ monomer, calcium ions, and lipid bilayer driving the monomer penetrate the POPC lipid bilayer. Surprisingly, we identified that Ca^2+^ ions inhibit the Aβ_1–42_ monomer penetration into the membranes; and prevent the contact between the DMPC and the Aβ_1–42_‐Cu^2+^ monomer due to a higher population of β‐hairpin at the C‐terminal and Phe20‐Ala30 residues. This evidence is consistent with the western blot experiment data^87^ by reporting two significant events: (i) Ca^2+^ mediates the Aβ_1–42_ monomer disrupt the DMPC bilayer through N‐ and C‐terminal interaction to the lipid headgroup and (b) Ca^2+^ does not encourage Aβ_1–42_ monomer to penetrate the membrane because Ca^2+^ forming bridge between the peptide and membrane, as shown in Video S1.

The attraction between the monomer and peptide is guided by two main driving forces (Video S1): the electrostatic interaction between positively charged Arg5 residue and negatively phosphate moieties of lipid headgroup and van der Waals interactions between hydrophobic residues of C‐terminal (Ile32‐Ile41) and the phosphate group. If the Aβ_1–42_ monomer is away from the DMPC bilayer, Ca^2+^ attraction mediates the monomer closer to the membrane. Subsequently, when the monomer is in contact with the membrane, the electrostatic and the van der Waals interactions have inhibited by calcium ions forming a bridge between the phosphate moieties and the peptides. As a result, Ca^2+^ significantly decreased the peptide‐membrane binding affinity and prevented monomer diffusion into the DMPC bilayer. In contrast, Ca^2+^ ions can inhibit the interactions between Aβ_1–42_‐Cu^2+^ monomer and the membranes, and Aβ_1–42_‐Cu^2+^ monomer exerts higher hydrophobicity, thus promoting the Aβ_1–42_‐Cu^2+^ aggregation at extracellular. In other words, Cu^2+^ binding and Ca^2+^ interaction to the monomer involved avoiding the interaction between the Aβ_1–42_ monomer and the DMPC bilayer.

To this end, Lockhart and Klimov^50^ performed REMD simulations to investigate the binding mechanism between the truncated Aβ_10–40_ monomer and the DMPC bilayer in the presence of calcium ions. They found that Ca^2+^ ions break the Asp23‐Lys28, compelled Lys28 to interact with the bilayer, and mediate to enhance the monomer–membrane interaction through electrostatic contacts between charged residues of the monomer and lipid polar headgroup. These are driving the monomer insertion into the membrane. In contrast, the results of our present simulations proved that the binding affinity between full‐length Aβ_1‐42_ and DMPC bilayer is significantly decreased by the influence of Ca^2+^ ions that prevent penetration into the membrane. Notably, Arg5 residue is strongly involved in the binding with the lipid headgroup of DMPC, and this residue is not placed in the truncated monomer. These results imply that the binding character of the truncated Aβ_10–40_ and full‐length of Aβ_1–42_ with the zwitterionic DMPC bilayer are different from each other.

## 5 CONCLUSION

We have performed microsecond molecular dynamics simulation to explore the intriguing feature of the full‐length of Aβ_1–42_ and Aβ_1–42_‐Cu^2+^ monomer with close contact with the DMPC bilayer in a higher concentration of Ca^2+^ ions. In this context, we reached eight conclusions:

1. In the case of Aβ_1–42_, the R4 region possessed higher helix content favoring the interaction to the DMPC bilayer and transformed to the β‐sheet conformation upon Cu^2+^ binding is not involved in interaction with the bilayer;
2. Cu^2+^ binding enhancing stronger Glu22‐Lys28 and Asp23‐Lys28 salt‐bridge stability promote turn conformation in R3 regions assisting to beta‐hairpin conformation at residues Phe20‐Ala30 in Aβ_1–42_ monomer.
3. Free energy analysis predicted local minimum conformations, where Cu^2+^ binding mediate Aβ_1–42_ monomer exerts higher solvent accessible surface area representing higher hydrophobicity character led to the aggregation‐prone structure.
4. In the case of Aβ_1–42_, Arg5 in R1 and Ile31, Leu34‐Val36, Gly38‐Ile41 in R4 regions are formed electrostatic interactions and van der Waals interactions to phosphate moieties of lipid headgroup are missing in the case of Aβ_1‐42_‐Cu^2+^.
5. In the case of the absence of calcium ions, N‐terminal residues of Aβ_1–42_ deeply penetrate from the surface to the center of the bilayer. In contrast to calcium ions presence, the N‐ and C‐terminal residues are involved only in surface contacts through binding phosphate moieties which confirmed by all regions of the peptide density mixed with the density mass of phosphorous atoms.
6. Aβ_1–42_‐Cu^2+^ actively participated in surface bilayer contacts in the absence of calcium ions. These contacts are prevented by forming a calcium bridge between Aβ_1–42_‐Cu^2+^ and the DMPC bilayer in the case of calcium ions presence, as confirmed by the density of Ca^2+^ ions, which was found higher between the peptide and lipid head group.
7. The total charge of Aβ_1–42_ is −3 attracted by the Ca^2+^ ions favor the monomer interaction with the membrane. In contrast, Cu^2+^ binding reduced the net‐charge of monomer to −1, which is attracted to Ca^2+^ was significantly decreased, preventing the Aβ_1–42_‐Cu^2+^ interaction to the membrane.
8. It is worth noting that both Aβ_1–42_ and Aβ_1–42_‐Cu^2+^ monomers undergo a random coil to β‐sheet transition in the aqueous phase and helix transition in the membrane phase.

In summary, all the findings demonstrate that electrostatic and van der Waals interactions are the principal driving forces in forming the interaction between the Aβ_1–42_ monomer and the membrane. As soon as the peptide‐membrane contact is formed, Ca^2+^ forms bridges between the monomer and the membrane since the Ca^2+^ ions are attracted by the phosphate moieties, resulting in the reduced peptide‐membrane binding affinities leading to preventing insertion of the Aβ_1–42_ monomer into the DMPC bilayer.

In this work, we presented the relationship between the Aβ monomers and the membranes; we expected that the oligomer form of Aβ_1–42_ and Aβ_1–42_‐Cu^2+^ could contact the biological membrane, possibly causing the polarization of the membrane links, leading to neuronal membrane damage. The latter seems to play a vital role in AD development; therefore, such studies are currently undergoing in my laboratory by combining coarse‐grained with all‐atom MD simulation^88^ in the ~50 μs range.

## AUTHOR CONTRIBUTIONS

Subramanian Boopathi performed the research, analyzed the data, and wrote the manuscript. Ramón Garduño‐Juárez reviewed, edited, and made grammar corrections. All authors have read and agreed to the published version of the manuscript.

## CONFLICT OF INTEREST

The authors declare no conflict of interest.

### PEER REVIEW

The peer review history for this article is available at https://publons.com/publon/10.1002/prot.26403.

## Supporting information


**Appendix S1** Supporting Information

**Video S1** Supporting Information

## Data Availability

The data that support the findings of this study are available in the supplementary material of this article.
