# Supplementary material for: Calcium inhibits penetration of Alzheimer's Aβ1–42 monomers into the membrane
Source: Proteins. 2022 Aug 10;90(12):2124–43. doi: 10.1002/prot.26403 (PMC9804374; doi:10.1002/prot.26403)
Supplement: Supplementary file 1 — Appendix S1 Supporting Information [file PROT-90-2124-s002.docx]

**Calcium Inhibits Penetration of Alzheimer’s Aβ_1-42_ Monomers into the Membrane**

**Subramanian Boopathi* and Ramón Garduño-Juárez***

**Instituto de Ciencias Físicas, Universidad Nacional Autónoma de México, Cuernavaca 62210, México.**

**Corresponding Authors’ names:**

**Dr. Subramanian Boopathi**

**Email:** [**boopathi@icf.unam.mx**](mailto:boopathi@icf.unam.mx)

**Prof. Ramón Garduño-Juárez**

**Email:** [**ramon@icf.unam.mx**](mailto:ramon@icf.unam.mx)

**This supporting information contains:**

**Figure S1 to S6,**

**Perl script for contact map and free energy landscape analysis,**

**Tables S1 and S2.**

**
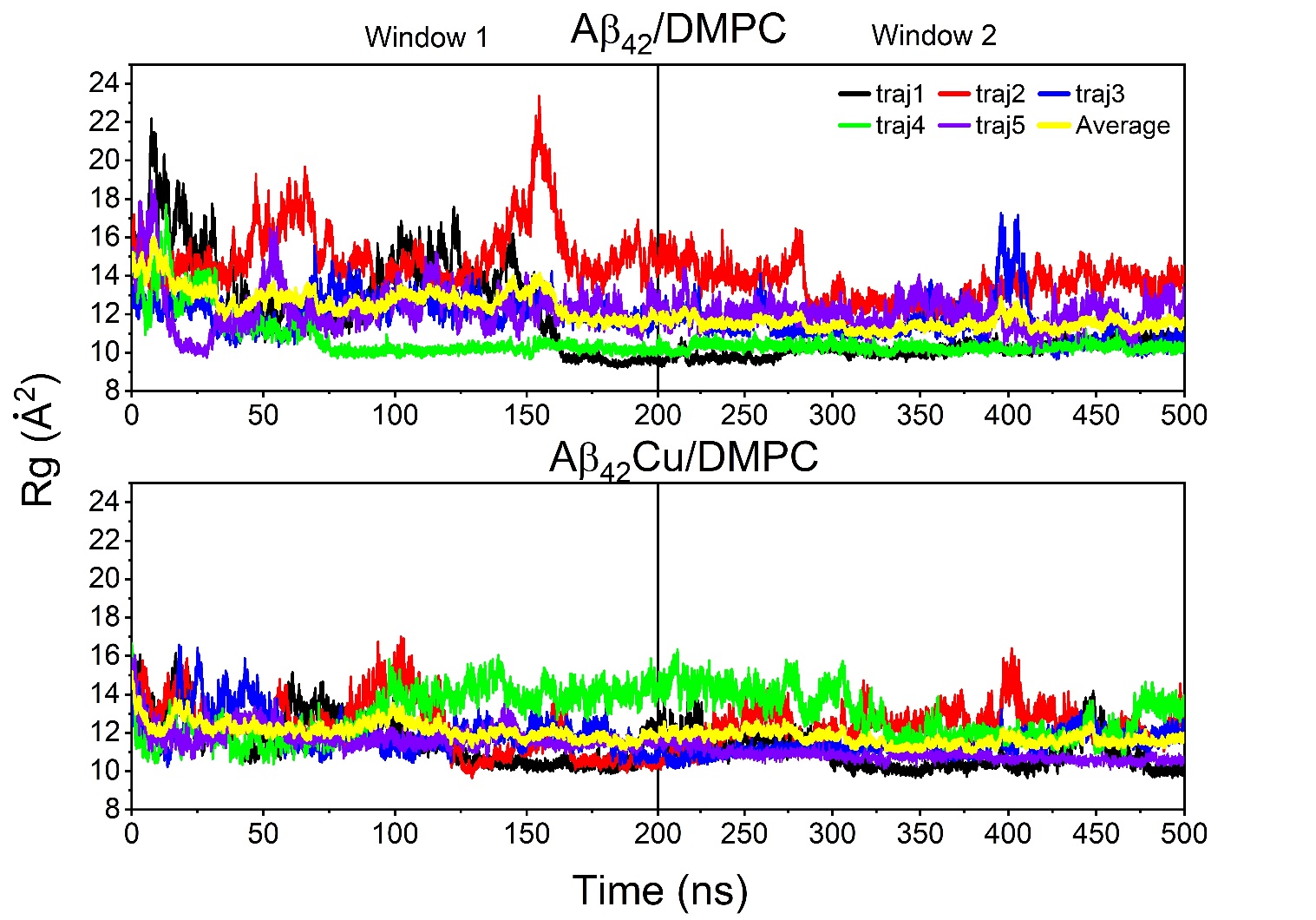
**

**A**

**
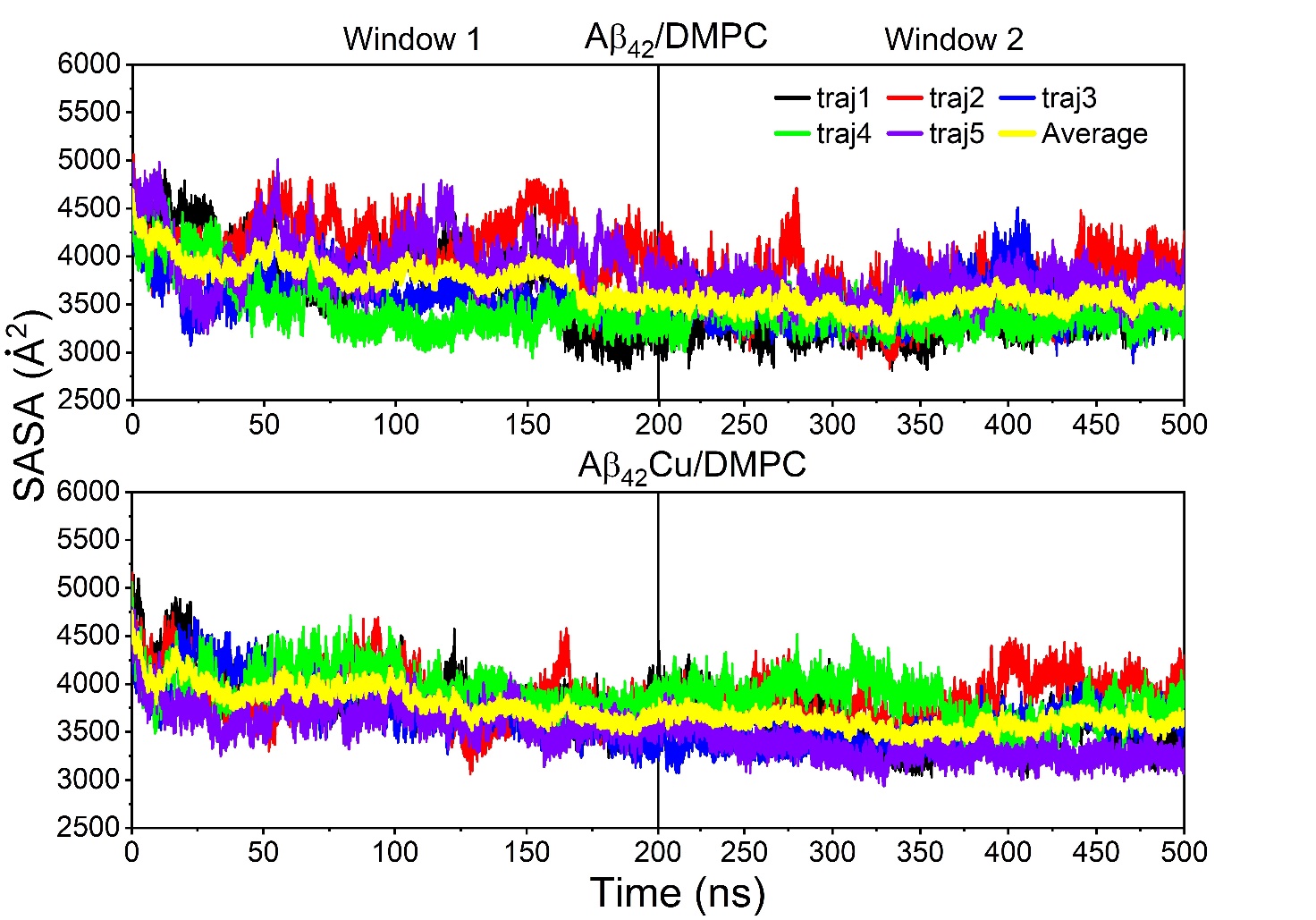
**

**B**

**
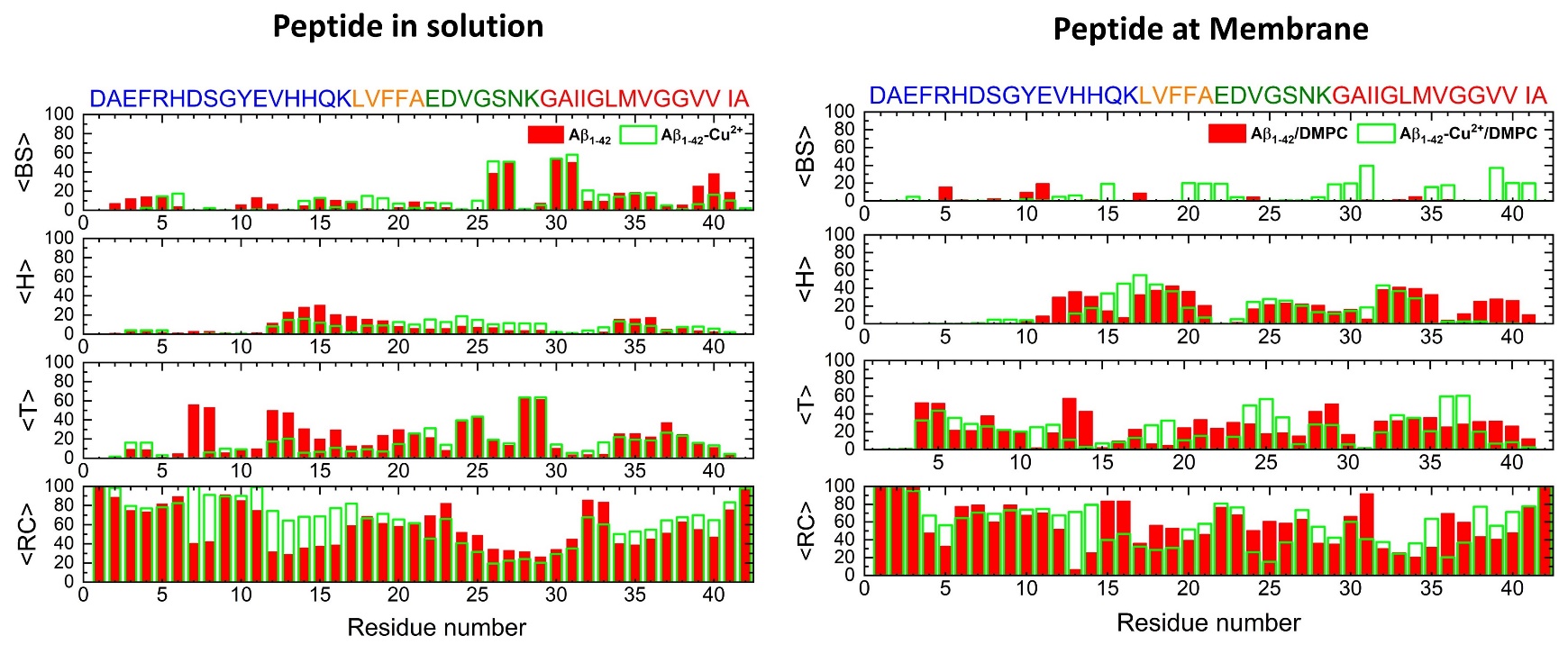
**

**C**

**D**


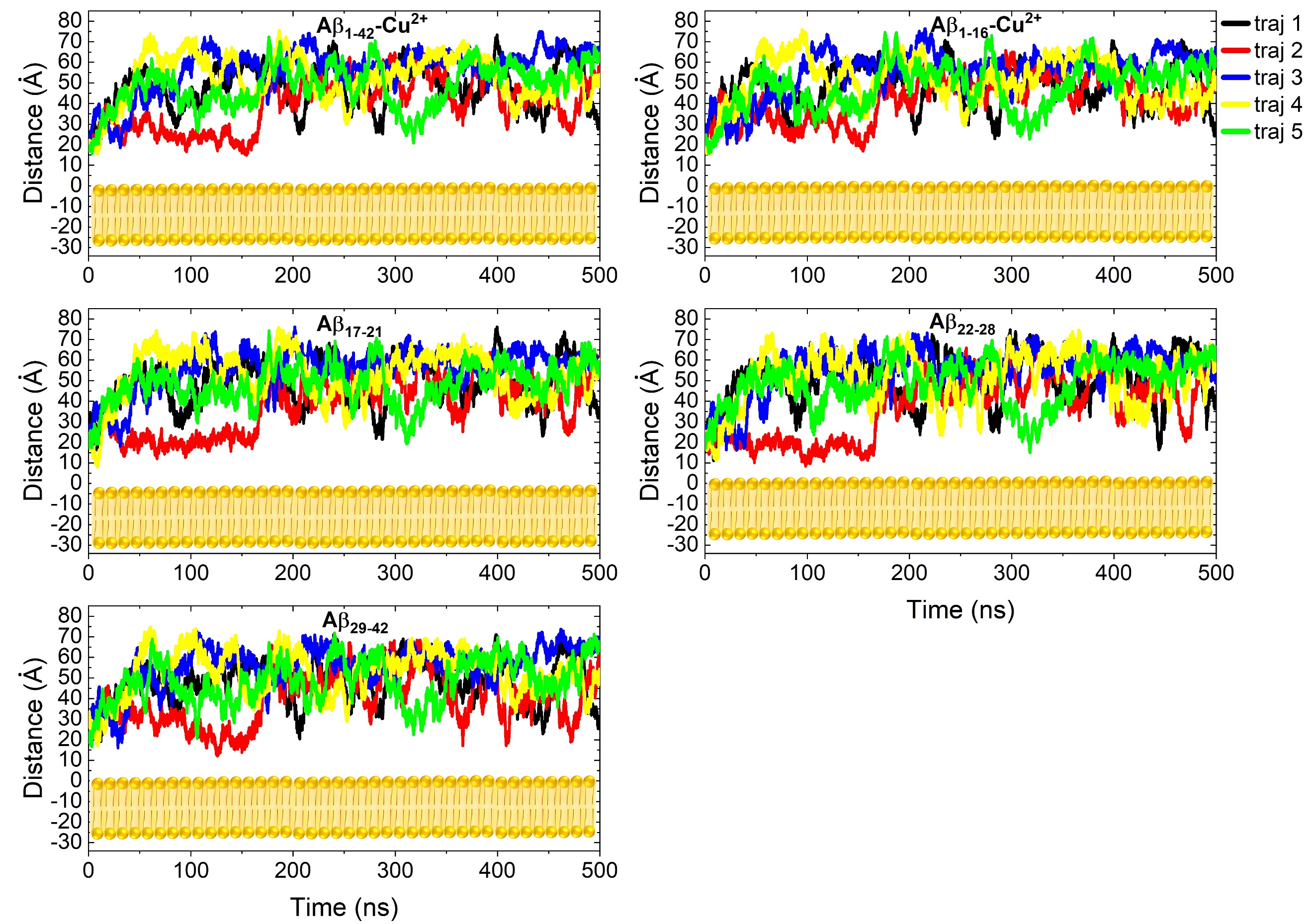


**Figure S1**: a) Radius of gyration (Rg) and b) Solvent Accessible Surface Area (SASA) were plotted against time. C) Residue-wise population distribution (%) of the secondary structure of Aβ_1-42_ and Aβ_1-42_-Cu^2+^ in the absence and presence of DMPC and Ca^2+^ ions. Analysis was carried out for an average of all trajectories, each acquired between 200 and 500 ns. A single letter code gives the peptide sequence at the top of the figure; blue, orange, green, and red are the R1, R2, R3, and R4 regions. Beta sheet, Helix, Turn, and Beta-sheet contents are represented by <BS>, <H>, <T>, and <RC>, respectively. D) Distance between center of mass of distance between full-length of Aβ_1-42_-Cu^2+^ and DMPC bilayer, different regions of the same peptide and DMPC bilayer plotted against time. The distance average between the peptide and membrane is greater than 10Å for all trajectories except trajectory 2 which involve weaker interaction since the distance average is about 7.5 Å during the first 150 ns. A larger distance implies that the probability of contact is very small.


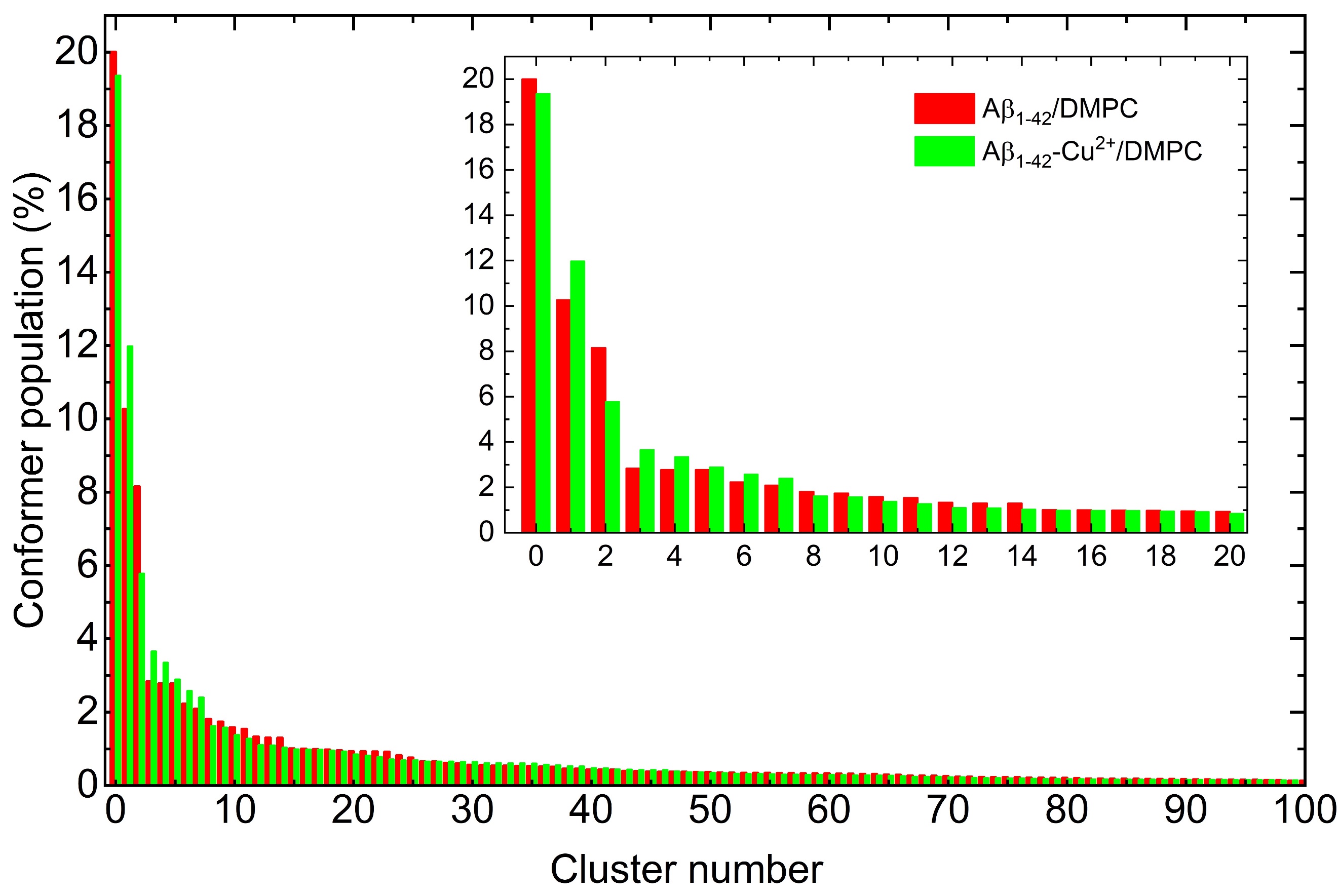


**Figure S2**: Cluster population for Aβ_1-42_ and Aβ_1-42_-Cu^2+^. Top twenty-cluster populations shown in inset figure.


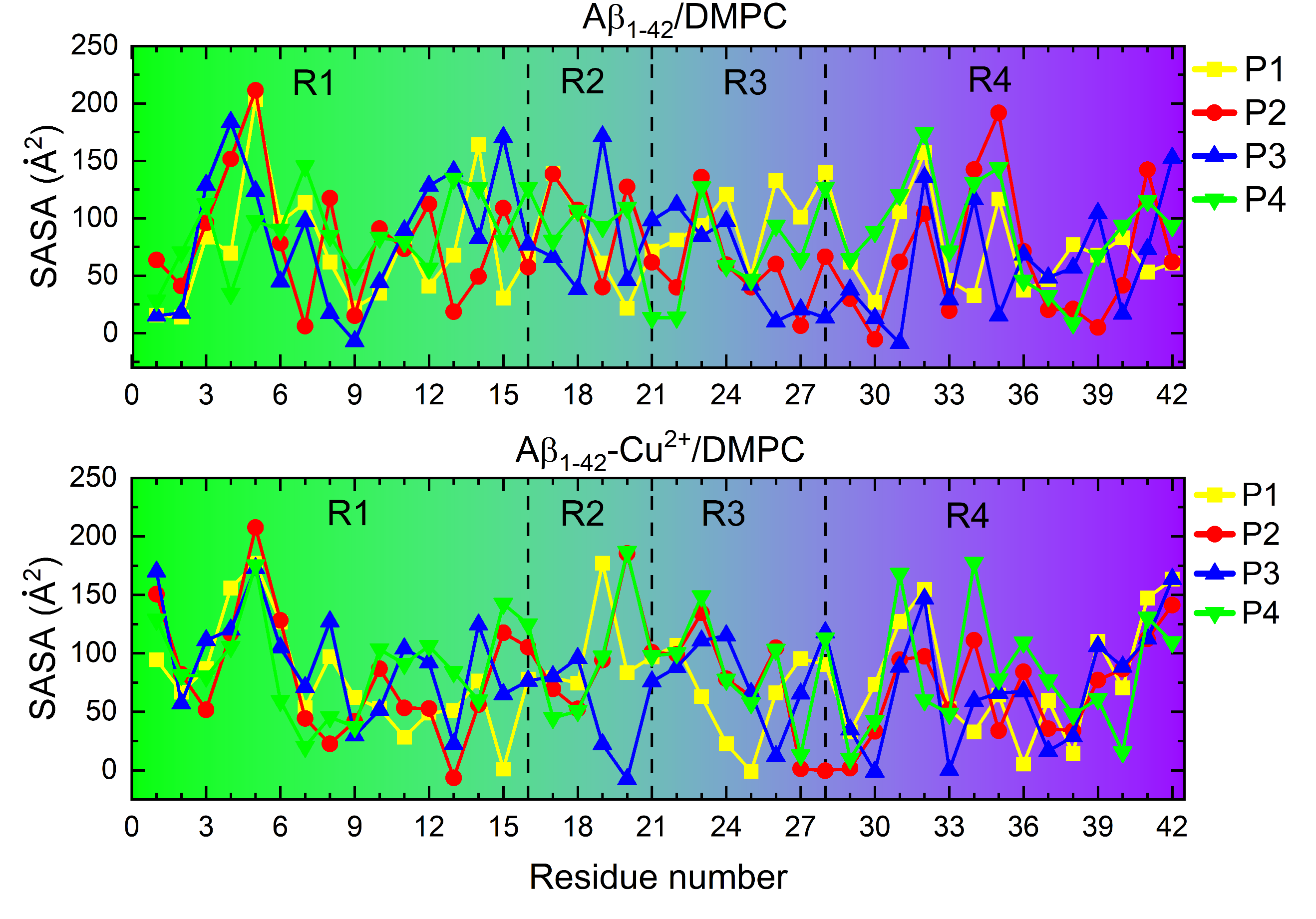


**Figure S3**: Residue-wise Solvent Accessible Surface Area (SASA) for P1, P2, P3 and P4 representative conformation at each local minimum basins shown in Figure 9.


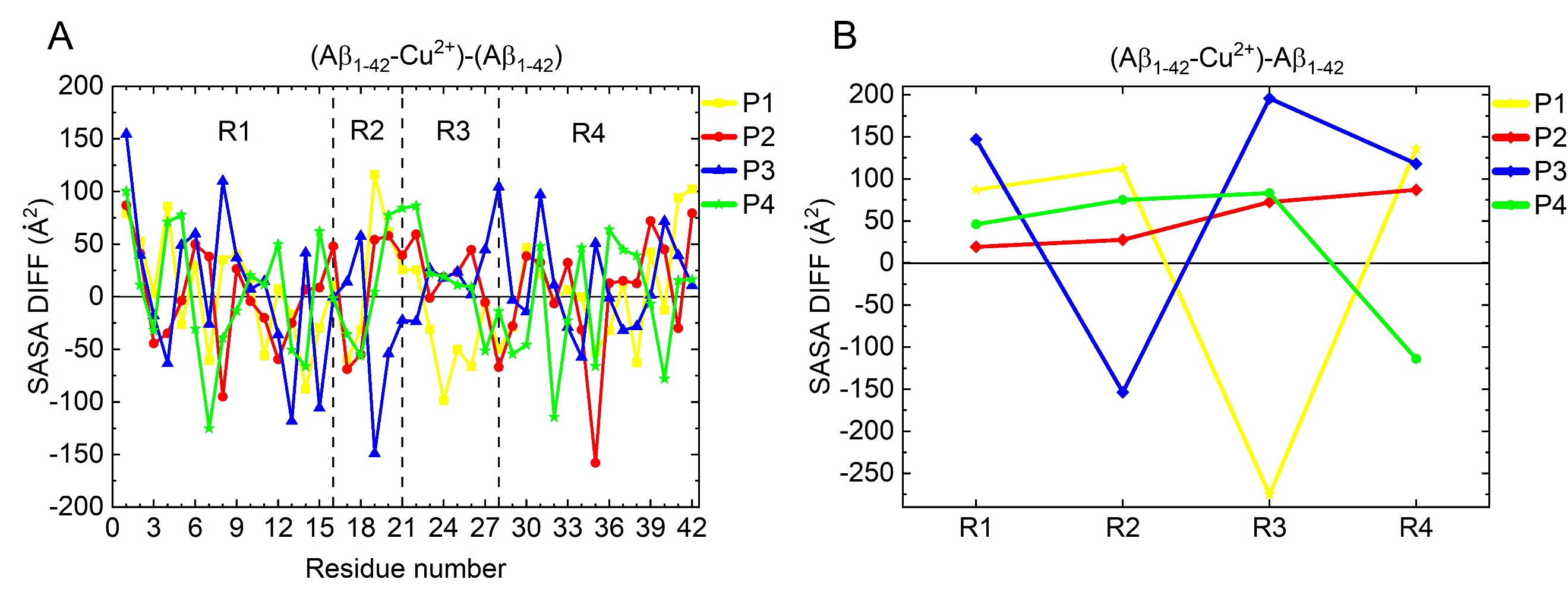


**Figure S4**: a) Residue-wise SASA differences between free and copper bound Aβ_1-42_ peptide; b) Region-wise SASA differences between Aβ_1-42_ and Aβ_1-42_-Cu^2+^ for the four representative structures identified in Figure 9.


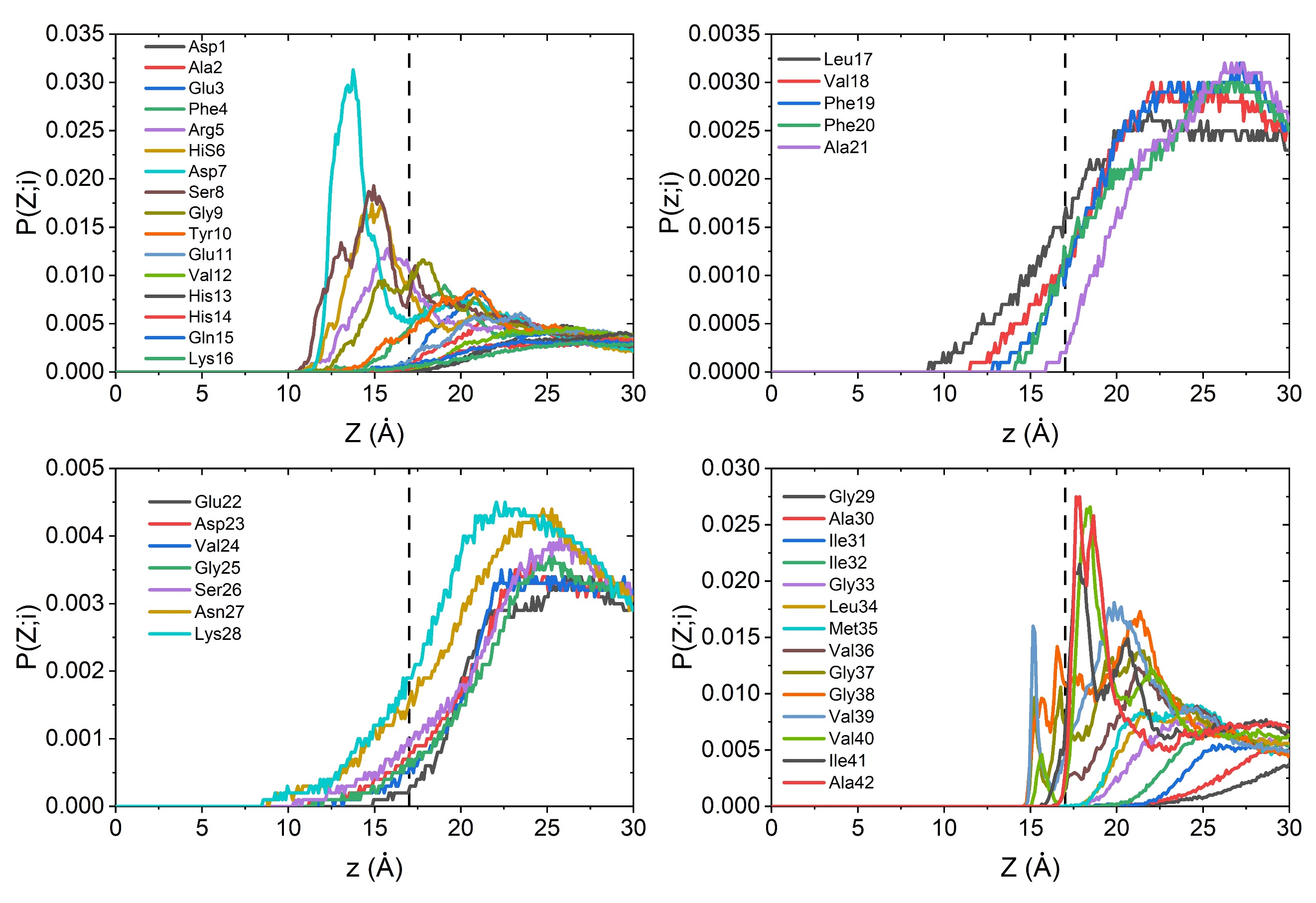


**Figure S5**: Probability distribution P (Z; i) of the i^th^ amino acid along the z-axis to the bilayer. The plot shows residues at N-terminal (amino acids 1-16), Central hydrophobic (17-21), Loop (22-28) and C-terminal (29-42) regions; The average position of the center of mass of Phosphorus atoms at Zp = ~17 Å is represented by dashed line.


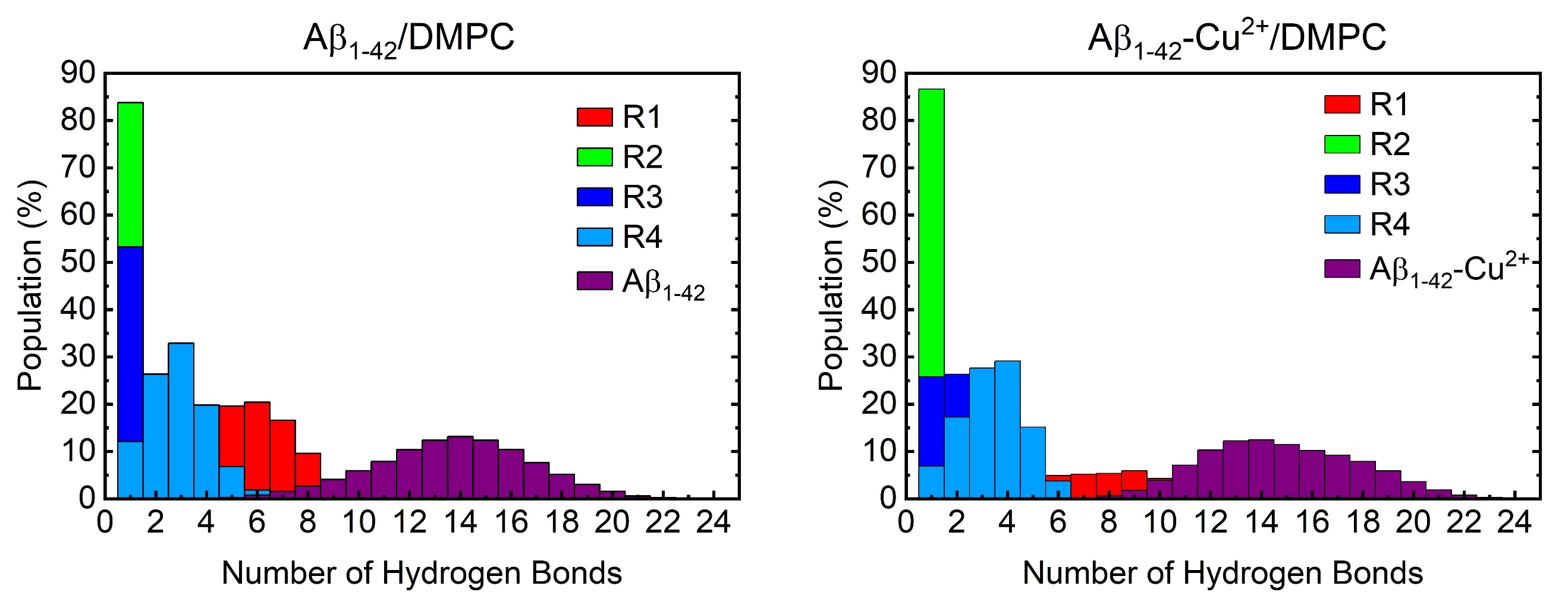


**Figure S6**: Averaged number of intrapeptide hydrogen bonds at different regions of full-length of Aβ_1-42_ and Aβ_1-42_-Cu^2+^ peptide. All ensembles at equilibrium were used to obtaining the result.

**Perl script for Contact Map Analysis**

#!/usr/bin/perl

$residuenumber=44;

$nsnapshots=200000;

for ($i=1;$i<$residuenumber;$i++){

for ($j=1;$j<$residuenumber;$j++){

$contact[$i][$j]=0;

}

}

for($k=1;$k<=200000;$k++){

$file="abctmol.pdb.$k";

for ($l=1;$l<=$residuenumber;$l++){

$sumx[$l]=0;

$sumy[$l]=0;

$sumz[$l]=0;

$natom[$l]=0;

}

open (FILE,"<$file");# read the r.pdb

while (<FILE>){

chomp;

# print "$_\n"; #print the line itself

#print "$.\n"; #print the line number ; \n means the line finished

@line=split(undef,$_); #array generate

if ($line[0].$line[1].$line[2].$line[3] eq "ATOM"){

$n=$line[6].$line[7].$line[8].$line[9].$line[10]; #atom number

$pdbatom[$n][1]=$line[32].$line[33].$line[34].$line[35].$line[36].$line[37]; # x axis of atom $n

$pdbatom[$n][2]=$line[40].$line[41].$line[42].$line[43].$line[44].$line[45]; # y axis

$pdbatom[$n][3]=$line[48].$line[49].$line[50].$line[51].$line[52].$line[53]; # z axis ...

$pdbatom[$n][4]=$line[22].$line[23].$line[24].$line[25]; # Residue number ...

# $totalatom+=1;

}

}

close FILE;

# Subroutine to calculate the distance between two atoms

sub dist(){

$distance=sqrt(($pdbatom[$_[0]][1]-$pdbatom[$_[1]][1])**2+($pdbatom[$_[0]][2]-$pdbatom[$_[1]][2])**2+($pdbatom[$_[0]][3]-$pdbatom[$_[1]][3])**2);

print "Distance between atom $_[0] and atom $_[1] is: $distance\n";

}

############################################################

sub com{

for (my $i=1;$i<=664;$i++){

$sumx[$pdbatom[$i][4]]+=$pdbatom[$i][1]; #xaxis

$sumy[$pdbatom[$i][4]]+=$pdbatom[$i][2]; #yaxis

$sumz[$pdbatom[$i][4]]+=$pdbatom[$i][3]; #zaxis

$natom[$pdbatom[$i][4]]+=1;

}

}

&com;

for ($i=1;$i<$residuenumber;$i++){

for ($j=1;$j<$residuenumber;$j++){

$var1=$i;

$var2=$j;

$x=$sumx[$var1]/$natom[$var1];

$y=$sumy[$var1]/$natom[$var1];

$z=$sumz[$var1]/$natom[$var1];

$x1=$sumx[$var2]/$natom[$var2];

$y1=$sumy[$var2]/$natom[$var2];

$z1=$sumz[$var2]/$natom[$var2];

$distance = sqrt(($x-$x1)**2+($y-$y1)**2+($z-$z1)**2);

if($distance<=6.5)

{

$contact[$i][$j]++;

}

}

}

}

##################################################################

open (RESULT,">contmapabmolCT.dat");

for ($i=1;$i<$residuenumber;$i++){

for ($j=1;$j<$residuenumber;$j++){

my $t=$contact[$i][$j]/$nsnapshots*100;

print RESULT "$i $j $t\n";

}

}

close RESULT;

**Perl Script for Free Energy Landscape Analysis**

#!/usr/bin/perl

$file="ab42rr.dat";

$nx=200;

$ny=200;

$fileout="pm3dscript1_d200_200.dat";

open (IN,"<$file");

$xmax=0;$xmin=1000;

$ymax=0;$ymin=1000;

while (<IN>){

chomp;

my @line=split;

if ($line[0] > $xmax ){$xmax =$line[0];}

if ($line[0] < $xmin ){$xmin =$line[0];}

if ($line[1] > $ymax ){$ymax =$line[1];}

if ($line[1] < $ymin ){$ymin =$line[1];}

}

close IN;

$dx=($xmax-$xmin)/$nx;

$dy=($ymax-$ymin)/$ny;

print "

xmax: $xmax

xmin: $xmin

dx: $dx

ymax: $ymax

ymin: $ymin

dy: $dy

";

$B=0;

open (OUT,">$fileout");

for (my $x=0;$x<$nx;$x++){

for (my $y=0;$y<$ny;$y++){

open (IN,"<$file");

while (<IN>){

chomp;

@line=split;

if ($line[0] >= $xmin+($x*$dx) && $line[0] < $xmin+($x+1)*$dx){

if ($line[1] >= $ymin+($y*$dy) && $line[1] < $ymin+($y+1)*$dy){

$B++;

}

}

}

close IN;

printf OUT ("%10.8f %10.8f %5s\n", $xmin+$x*$dx, $ymin+$y*$dy, $B);

$B=0;

}

print OUT "\n";

}

close OUT;

#########################################

#!/usr/bin/perl

$max_population=276;

$T=300;

open (PO,"<pm3dscrip2_200_200.dat");

while (<PO>){

chomp;

@line=split;

if (/^\s*$/){print "\n";}

elsif ($line[2] eq 0) {printf ("%10.4f %10.4f %10.4f\n",$line[0],$line[1],-0.0019872041*$T*(log(0.0001)-log($max_population)));}

else {printf ("%10.4f %10.4f %10.4f\n",$line[0],$line[1],-0.0019872041*$T*(log($line[2])-log($max_population)));}

}

close PO;

**Computational details:**

Our group (J. Phys. Chem. B 2017, 121, 4341-4354) has simulated the full-length of the Aβ1-42 peptide through two windows; first-run duration t1 (500 ns) and second-run duration are t2=2t1 (1000 ns). The secondary structure calculation was carried out for the two-time windows t1 and t2. They obtained secondary structure content at 311 K; the beta-sheet, helix, turn, and coil contents of the Aβ1-42 peptide in the two-time windows are the same. In our previous investigation, we studied the effect of Cu and Zn ions on the dynamics of Aβ1-42 for a 500ns simulation run. We predicted the most populated Aβ1-42 peptide conformers from the free energy landscape (Proteins. 2020, 88, 1285-1302) analysis, which are well-matched with the experimental results of Roychaudhuri (J. Mol. Biol. 2013, 425, 292-308), and beta-hairpin conformation observed at Gly25-Met35 is in good agreement with the results of Joan Emma Shea group (Biophys. J 2006, 91, 1638-1647). Due to the above evidence, we perform five trajectories of 500 ns productions run, a total of 2.5 µs carried out for the complex system.

Philippe Derreumaux’s (J. Phys. Chem. B 2017, 121, 5977-5987) group performed 144 µs replica exchange molecular dynamics simulation for Aβ dimer; they determined the equilibrium ensembles by using four force fields OPLSAA, CHARMM22*, AMBER99sb-ildn, and AMBERsb14 force fields with the TIP3P water model. They predicted AMBERsb14 and CHARMM22* ensemble overestimating the Circular Dichroism (CD)-derived helix content, and the OPLS-AA and AMBER99sb-ildn secondary structure contents, including beta-sheet contents, in agreement with CD data. They demonstrated that AMBER99SB-ILDN is a good estimator tool for the disordered protein but not for the folded protein due to overestimating helix content; therefore, AMBER99SB-ILDN can accurately predict beta-sheet contents as found in the CD- study. Since Amber14sb/TIP3P is similar to Amber99SB*-ILDN/TIP3P, above mentioned pieces of evidence strengthen our selected candidate that deserves to study the full-length of disordered Aβ1-42 peptide.

We have used bonded model of the full-length of Aβ1-42-Cu2+ taken from our group paper (ACS Chem Neurosci 2016, 7, 10, 1348-1363), where Cu2+ coordinated with the nitrogen and oxygen atoms of Asp1, Nδ of His6, and Nε of His13 geometry optimizes (Table S1). Their binding distances are around 2.0Å, similar to previous experimental and theoretical results (JACS 2011, 133, 3390-3400; JPC B 2014, 118, 4840-4850; J Biol Inorg 1012, 17, 927-938). This coordination mode was close to the physiological pH of 6.9. Pham et al. (ACS Chem Neurosci 2016, 7, 10, 1348-1363) have reported force-field parameters between Cu2+ and the coordination atoms used in our present work.

We compared the secondary structural results for an averaged five independent trajectories over the two-time windows [200, 500 ns] and tabulated in table S2 that reported Cu^2+^ binding could decrease the helical structure and increase the beta-sheet in the Aβ peptide, leading to a decrease in the contacts with the membranes since helical structure more favor membrane surface interactions. This result conjectured that Ca^2+^ ions mediated Aβ_1-42_ contacts with the membrane were prevented by Cu^2+^ binding to the peptide.

**Table S1**: Force constants for the Cu^2+^ bonded Aβ_1-42_ complex.

| Bond | Bond Length (Å) | Force Constant (Kcal/mol. Å^2^) |
| --- | --- | --- |
| Cu-O(Asp1) | 2.03 | 50.12 |
| Cu-N(Asp1) | 2.02 | 75.27 |
| Cu-Nδ(His6) | 2.02 | 70.73 |
| Cu-Nε(His13) | 2.04 | 61.69 |
| Lennard-Jones | R_min_/2 (Å) | ε (Kcal/mol) |
| Cu | 1.20 | 0.05 |

**Table S2**: Secondary structure contents (%) ± standard error for five independent trajectories simulation run.

|  |  | Helix | Beta | turn | coil |
| --- | --- | --- | --- | --- | --- |
| Aβ1-42/DMPC | Window 1 | 12.75 ± 2.58 | 1.31 ± 0.39 | 21.71 ± 1.60 | 64.21 ± 3.10 |
|  | Window 2 | 16.57 ± 0.87 | 1.71 ± 0.84 | 23.46 ± 1.15 | 58.19 ± 1.13 |
| Aβ1-42Cu/DMPC | Window 1 | 9.20 ± 1.95 | 5.25 ± 1.16 | 20.33 ± 1.89 | 65.20 ± 2.02 |
|  | Window 2 | 13.39 ± 2.06 | 7.02 ± 2.50 | 21.08 ± 3.33 | 58.52 ± 3.09 |
